# Global transcription network incorporating distal regulator binding reveals selective cooperation of cancer drivers and risk genes

**DOI:** 10.1093/nar/gkv532

**Published:** 2015-05-22

**Authors:** Kwoneel Kim, Woojin Yang, Kang Seon Lee, Hyoeun Bang, Kiwon Jang, Sang Cheol Kim, Jin Ok Yang, Seongjin Park, Kiejung Park, Jung Kyoon Choi

**Affiliations:** 1 Department of Bio and Brain Engineering, KAIST, Daejeon 305-701, Republic of Korea; 2 Samsung Genome Institute, Samsung Medical Center, Seoul 135-710, Republic of Korea; 3 Korean Bioinformation Center, KRIBB, Daejeon 305-806, Republic of Korea

## Abstract

Global network modeling of distal regulatory interactions is essential in understanding the overall architecture of gene expression programs. Here, we developed a Bayesian probabilistic model and computational method for global causal network construction with breast cancer as a model. Whereas physical regulator binding was well supported by gene expression causality in general, distal elements in intragenic regions or loci distant from the target gene exhibited particularly strong functional effects. Modeling the action of long-range enhancers was critical in recovering true biological interactions with increased coverage and specificity overall and unraveling regulatory complexity underlying tumor subclasses and drug responses in particular. Transcriptional cancer drivers and risk genes were discovered based on the network analysis of somatic and genetic cancer-related DNA variants. Notably, we observed that the risk genes were functionally downstream of the cancer drivers and were selectively susceptible to network perturbation by tumorigenic changes in their upstream drivers. Furthermore, cancer risk alleles tended to increase the susceptibility of the transcription of their associated genes. These findings suggest that transcriptional cancer drivers selectively induce a combinatorial misregulation of downstream risk genes, and that genetic risk factors, mostly residing in distal regulatory regions, increase transcriptional susceptibility to upstream cancer-driving somatic changes.

## INTRODUCTION

Transcription networks provide a basis for the understanding of regulatory mechanisms underlying particular biological processes or traits (1–3). In particular, non-coding genetic variations may contribute to relevant phenotypes by disturbing the transcription network (4–6). In cancer, the effect of non-coding risk factors (SNPs) on transcription perturbation has received scholarly attention recently (7,8). However, our understanding of the mechanisms by which such regulatory variations act at the systems level is limited due, at least in part, to incomplete modeling of gene regulatory networks. Although more genome-wide data for transcription factor (TF) binding are becoming available, identifying true targets is complicated when TF binding is observed at distal regulatory elements (DREs) because only a small fraction of DREs interact with the nearest transcript (9), while most TFs preferentially bind to DREs rather than proximal regulatory elements (PREs) (10–14). Numerous DRE–PRE and PRE–PRE interactions have been reported (15–17).

Moreover, physical TF-gene couplings do not necessarily support functionality or reveal directionality (i.e. activation or repression). TF knockdown does not necessarily change the expression levels of genes that are physically bound as observed in yeast (18) and human (19). The problem is exacerbated when sequence-based inferences of TF binding are used because the presence of recognition motifs does not necessarily indicate actual binding. Motif-based inferences can be tested based on correlation in the gene expression of the putative regulators and target genes (4). However, transcriptional causality cannot be confirmed solely by expression correlation. A promising approach for identifying functional TF target genes is Bayesian network modeling of causal relationships embedded in gene expression patterns (3,20,21). Moreover, the use of gene expression data from clinical tissues may complement data obtained using cultured cells. However, the large computational burden limits the scalability of Bayesian modeling, which accounts for the lack of a global Bayesian network in humans to date.

In this work, we designed a comprehensive Bayesian prior model by leveraging the ENCODE data for TF binding and chromatin accessibility, TF motif information (22), three-dimensional chromatin interactomes (15,16) and gene expression QTL (eQTL) mapping (23) in breast cancer. We then tested the transcriptional causality of the prior interactions by using ∼1400 breast cancer microarray and RNA-seq profiles from The Cancer Genome Atlas (TCGA). We also developed a computational method to construct for the first time a global network in humans. These enabled a comprehensive gene-wise characterization of cancer-related DNA variants at an unprecedented level of biological coverage and accuracy. Our primary focus was to understand functional regulatory connections among genes containing predicted driver mutations in coding regions (24–26) and genes associated with cancer risk factors discovered by genome-wide association studies (GWASs) (27).

## MATERIALS AND METHODS

### Data sets used for network construction

For breast cancer network construction, TF ChIP-seq experiments in 104 different cell lines, including the breast cancer cell lines MCF-7 and T47D, were obtained from the ENCODE project database. In addition, the position weight matrix of the TransFac (28) was mapped to DHS positions in the two breast cancer cell lines (MCF-7 and T47D). Among 485 TFs that were present in either the ChIP-seq or motif data set, 436 were associated with DHSs in the breast cancer cell lines and were also covered by the TCGA breast cancer gene expression data. Chromatin interactions mediated by RNA polymerase II in MCF-7 cells (16) were incorporated into our TF and eQTL prior probabilistic model. For Bayesian learning of causal network structure, the TCGA microarray and RNA-seq data encompassing 1386 tumor samples were obtained, normalized and discretized. For leukemia network construction, we used large-scale gene expression data (29,30) and ENCODE epigenomics data in K562 including chromatin interactions via RNA polymerase II (16).

### Network construction

The possible scenarios of TF binding via DRE–PRE and/or PRE–PRE chromatin interactions were used to construct a probabilistic prior model. We merged the TF and eQTL prior relationships by choosing the maximum prior probability for duplicate pairs of regulators and targets. The functional causality of the prior interactions was tested based on our Bayesian network learning procedures that coupled the GA with the MCMC-based algorithm. Instead of using random seed networks, we applied the GA to obtain 1000 suboptimal networks, each of which was evolutionarily selected from 128 initial candidates and then used as the input of the MCMC-based learning. The following is the overall procedure of our GA-based selection of suboptimal network structures. First, the initial population of chromosomes (networks) was created by adding links based on the prior probability table and then adding randomly generated links based on the Poisson distribution. Second, the evolution loop was run by evaluating each chromosome based on the fitness score function, classifying the chromosomes into four groups according to their fitness score, performing the crossover of chromosomes based on their group identity, generating mutations according to the mutation rate defined for each group, and repeating the above steps for a predefined maximum number of generations based on stopping criteria. Third, the chromosomes were combined and the optimal network was output. Fourth, the above steps were repeated to generate a predefined number of different networks. Finally, the different networks were combined by selecting the links that appeared commonly in a defined number of networks. To assess the effect of our prior data on the performance of Bayesian learning and the composition of output networks, we used prior subsets or perturbed our prior table, and traced the convergence of the fitness score and the number of the recovered edges over 20 000 GA generations. Further details are provided in Supplementary Information. The prior framework and global network are available at our web page (http://omics.kaist.ac.kr/resources/).

### Prior evaluation

We first evaluated our prior information using a previously established Bayesian method (3,20,21,31–33). To do so, we chose a test co-expression module. WGCNA, an R package for weighted correlation network analysis (34), and the Dynamic Tree Cut library for R (35), were used to unambiguously identify co-expression modules. Based on ∼1400 TCGA breast cancer gene expression profiles, five major co-expression modules were identified. Two of them were significantly enriched for genes involved in breast cancer, cell cycle, DNA replication and DNA damage in terms of Gene Ontology. These two modules were combined and used to construct four different test sub-networks, each based on distal and proximal TF binding, proximal TF binding only, eQTL, or random selection following the Poisson distribution. For each node in each of the four test networks, we calculated the frequency of true positive (TP), false negative (FN) and false positive (FP) links by interrogating Reactome (http://www.reactome.org) (36), a manually curated and peer-reviewed functional interaction database. We also used a TF-target link catalog created by combining well-established open-access databases, namely, PAZAR (37) and HTRI (38). The F1 value was obtained as 2*TP/(2*TP+FP+FN) for outgoing links of each node, and the overall F1 frequency was compared for the four networks. For global network evaluation against the Reactome database, we used precision, TP/(TP+FP), instead of F1, because of disproportionately high FN caused by large network size and small database size. For global prior evaluation, we assessed the four partial prior tables in comparison with the full prior model during our GA-based network evolution. We traced the convergence of the fitness score and the number of the recovered edges over 20 000 GA generations in 10 independent runs.

### Subclass analysis

Gene expression data for a total of 103 tumor samples with receptor status information and matched normal tissue were obtained from the TCGA data portal. Based on the status of the three receptor proteins, namely, estrogen receptor (ER), progesterone receptor (PR) and human epidermal growth factor receptor 2 (HER2), we classified the samples as luminal A (ER positive and/or PR positive, HER2 negative), luminal B (ER positive and/or PR positive, HER2 positive), HER2-enriched (ER negative, PR negative, HER2 positive), or basal-like or triple-negative (ER negative, PR negative, HER2 negative). Subclass-specific genes were identified as differentially expressed between tumor and normal tissues by at least 2-fold in >20% of patients who were classified as that subclass.

### Drug response matrix

We obtained gene expression data for MCF7 cells in response to 155 small molecules (39). For each small molecule, we selected the 200 genes that exhibited the largest gene expression changes. For each of these responsive genes, the closest upstream TF was obtained. For each TF, a connectivity score was calculated for each small molecule as the fraction of the responsive genes that were connected to the given regulator. This process led to a matrix of connection scores between transcription regulators and small molecules. After column-wise and row-wise normalizations, unsupervised two-way hierarchical clustering was performed based on the Spearman's rank correlation and pairwise complete-linkage method. Small molecules whose mechanism of action is poorly understood and TFs whose normalized connectivity score was close to zero (−0.2–0.2) for every small molecule were removed, leading to a matrix with 85 TFs and 135 drugs.

### Expression perturbation

BRCA1 mutation status and gene expression data for the patients with mutation information available were obtained from the TCGA database. The expression perturbation of the BRCA1-downstream genes was compared between patient groups (BRCA1-mutated versus non-mutated). For each gene, the average expression perturbation of each group was obtained as

\begin{equation*} \frac{{\sum\nolimits_{i = 1}^n {|X_i - E|} }}{n} \end{equation*}

where *X_i_* is the expression level in the *i*th sample in the group, *n* is the number of samples in the group and *E* is the expected expression level of the gene. *E* was estimated as the mean expression of the gene across all available samples.

### Identification and analysis of transcriptional drivers and risk genes

We collected 519 breast cancer risk loci by including SNPs in linkage disequilibrium with the reported SNPs in the GWAS catalog (27). We first searched our global network for their targeting genes along with responsible regulators as indicated by our TF prior information. We then searched for the genes that were genetically associated with the risk loci by interrogating previous eQTL maps. Non-coding mutations in breast cancer were obtained from the whole-genome sequencing of 21 patients (40,41) and filtered by interrogating the catalog of polymorphisms among 1092 normal individuals (42) and the TransFac position weight matrix (28). The clinical mutation simulation was based on processed whole-genome sequencing data in leukemia, melanoma, glioma, and gastric, liver, kidney, lung, prostate, and colorectal cancers as obtained from the ICGC data portal or supplemental websites. Mutations were randomly selected from this pool and assigned to 23 virtual chromosomes in 21 virtual patients while maintaining the chromosomal and individual distribution identical to those of the breast cancer mutations. For the *in silico* simulation, chromosomal positions were randomly selected with the same distribution. Regulatory driver factors were identified as having a significantly large number of recurrently affected target genes (*P* < 0.05 from the *in silico* or clinical simulation). Two of the 17 regulatory driver factors were coding driver factors. In total, 44 transcriptional cancer drivers were identified. To calculate misregulation concordance, gene expression data for 170 tumor-normal matched samples were obtained from the TCGA data portal. For each gene, patient samples were assigned a value of −1 (down-regulated), 0 (not changed), or 1 (up-regulated) according to the differential expression between cancer and normal at the threshold of 2-fold. The absolute correlation coefficient between the differential expression vector of regulators and that of their target genes was examined. The mutation status of GATA3 in each patient was also obtained from the TCGA data portal, and its correlation with the differential expression of the target genes was calculated. To understand the functional associations between the drivers and risk genes, we used GeneMANIA (43) (http://www.genemania.org/) to identify enriched Gene Ontology terms for transcriptional drivers and their downstream risk genes with high misregulation concordance (*r* > 0.3). Significant enrichment (*q* < 0.1) was found for GATA3, FOXM1 and E2F1. Genotype information for the 170 tumor-normal matched patients was obtained from the TCGA data portal. Risk alleles were determined by comparing allele frequencies between the patient (TCGA) and normal (1000 Genomes) populations. Misregulation concordance was calculated as above for the pairs of the drivers and risk genes when >10 patients were present in both the risk and non-risk groups. The significance of misregulation concordance was determined for each group by the *P*-value of the correlation coefficient. Heterozygous samples were classified into the risk group.

## RESULTS

### Prior framework construction and its evaluation

**Figure 1.**
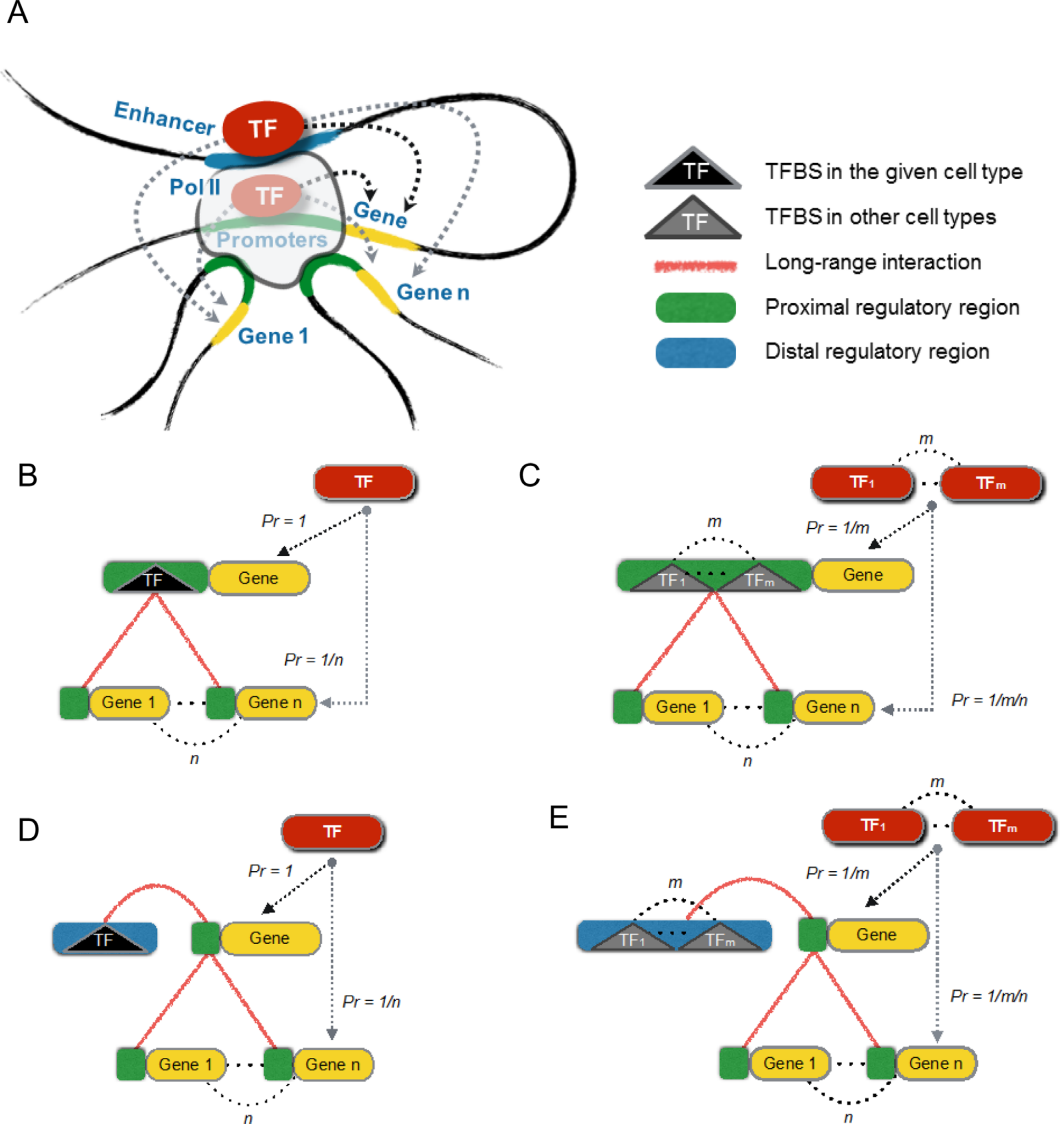
Bayesian prior model based on physical TF binding and long-range chromatin looping. (**A**) Schematic view of possible regulator-target interactions. (**B**) Model for PRE binding observed in breast cancer cells. (**C**) Model for PRE binding observed in other cell types but within accessible chromatin in breast cancer. (**D**) Model for DRE binding observed in breast cancer cells. (**E**) Model for DRE binding observed in other cell types but within accessible chromatin in breast cancer. (B–E) TF binding was defined either as the peak of ChIP-seq tags or as the presence of cognate motifs within DHSs.

Bayesian prior models were constructed based on TF binding, genetic association of gene expression and three-dimensional chromatin interactions. TF binding was defined either as the peak of chromatin immunoprecipitation (ChIP)-sequencing (ChIP-seq) tags or as the presence of cognate motifs within DNaseI hypersensitive sites (DHSs). The TF prior model (Figure 1) was developed in the present study, while the eQTL prior model (Supplementary Figure S1) was modified from previous Bayesian approaches (3,20,21). Details of the models are described in the Supplementary Information. A total of 2 960 981 relationships between 436 TFs and 11 932 putative target genes were incorporated into our TF prior model. We merged the TF and eQTL prior relationships while choosing the maximum prior probability for duplicate pairs of regulators and targets. This process yielded a total of 3 328 575 unique prior relationships between 9679 potential regulators and 13 026 putative target genes.

For prior evaluation, we first used an established Bayesian method. Markov Chain Monte Carlo (MCMC) has been used to model hundreds to thousands of genes in a predefined coexpression module (3,20,21,31–33). We followed this approach by first identifying a key coexpression module (Supplementary Figure S2) and constructing an MCMC-based Bayesian network for this module. Four different test networks were generated based on four different subsets of our prior information, namely, the complete TF priors (covering both DREs and PREs), the proximal TF priors (considering PREs only), the eQTL priors and the null prior set consisting of randomly generated links.

**Figure 2.**
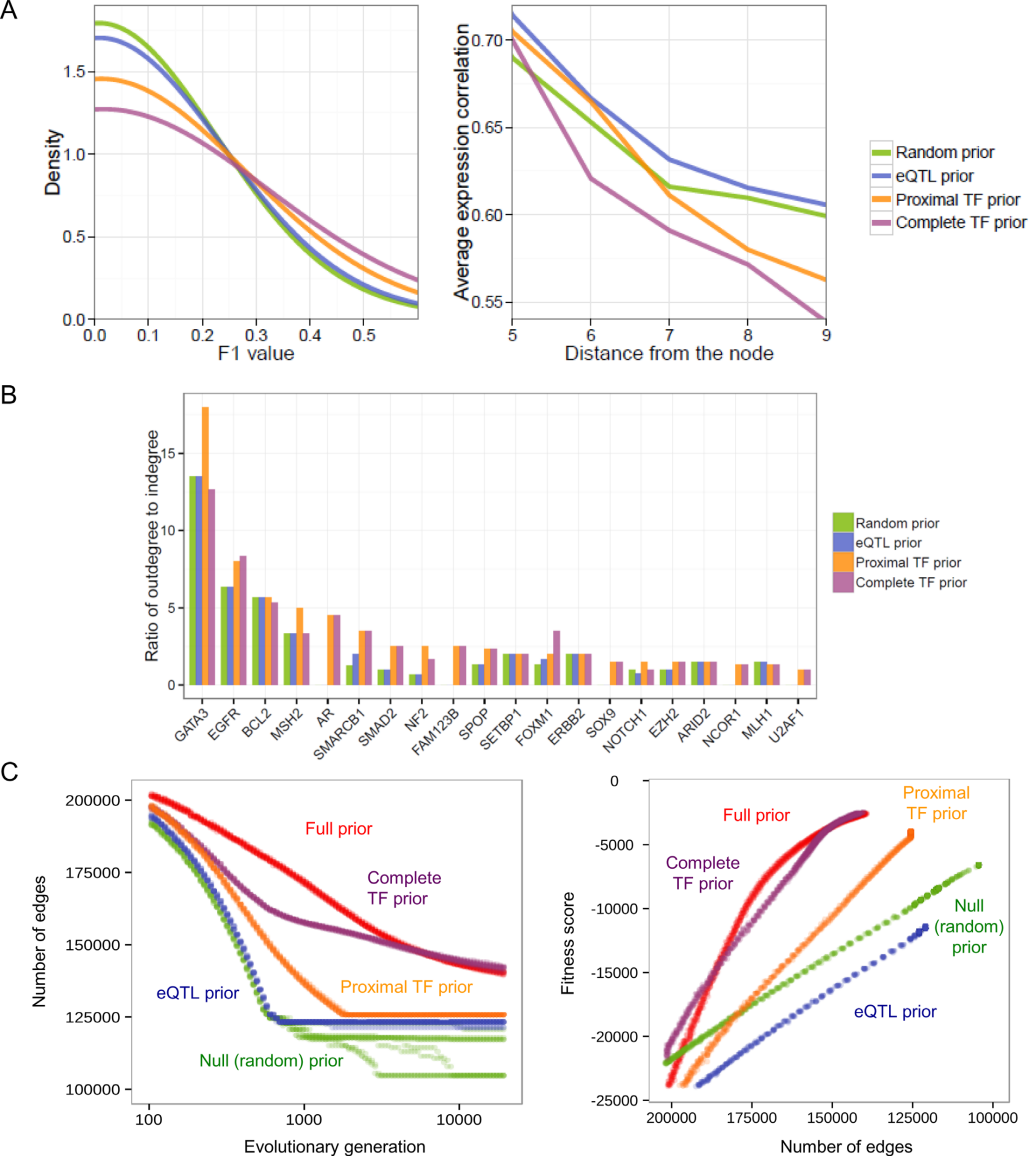
Quantitative prior evaluation. (**A**) Evaluation of four different test networks built on four different prior subsets. (Left) Distribution of the F1 scores for edges in a key breast cancer subnetwork as calculated by interrogating a manually curated and peer-reviewed pathway database. (Right) The average gene expression correlation of a node with other nodes at a varying network distance. (**B**) Ratio of the outdegree to indegree of TF nodes in the tested subnetworks. (**C**) Global network performance of four partial prior models. Convergence patterns were observed in 10 independent GA runs that used each prior subset by tracing the number of recovered edges according to the number of GA generations (left) and by tracing the fitness score according to the number of edges (right).

We computed the relative fraction of true positive links as the F1 score for each node by querying a manually curated functional interaction database and known TF-target relationship databases (see Methods). The distribution of the F1 scores was compared among the four test networks. As shown in the left panel of Figure 2A and Supplementary Figure S4A, the complete TF prior model led to a network with the largest number of high-F1 links and lowest number of low-F1 links, indicating that modeling long-range TF interactions is essential for accurately recovering true functional interactions. Moreover, the TF prior models were less dependent on gene expression patterns than the null prior model as assessed by expression correlations between distant nodes in the network (right panel of Figure 2A). By contrast, the eQTL priors as well as the random priors did not add a substantial amount of additional information beyond the expression patterns. Prior information also appeared to affect network topology. The relative distribution of the outdegree and indegree of TFs in the tested sub-network differed depending on which prior subset was used (Figure 2B).

### Global network construction and prior evaluation

To enable a global regulatory network, we coupled a genetic algorithm (GA) with MCMC. We first applied the GA to obtain 1000 suboptimal networks, which were fed into the MCMC pipeline (summarized in Supplementary Figure S3A), thereby achieving a >120-fold increase in the initial search pool and a >22-fold reduction in computation time compared with the pure MCMC approach (see Supplementary Information). We attempted to compare the outputs of our GA-MCMC hybrid approach and the pure MCMC method. Because the pure MCMC was practically impossible to implement for full-scale global network construction, we performed a pilot GA-MCMC and pilot pure MCMC by using 10 instead of 1000 MCMC seed networks based on the identical prior data and entire gene expression data. There was 93.2% consistency between the two output networks (Supplementary Figure S3B).

Based on the ∼1400 TCGA expression data used for causal network construction, the correlation of regulator expression and target expression was obtained for each link functionally recovered from the TF prior data. Activation and inhibition regulator-target relationships were identified based on the sign of the correlation when the absolute correlation coefficient was greater than 0.1. Without regard to the binding mode of the TF prior interactions, activation was observed approximately 1.5-fold more frequently than inhibition.

We first evaluated the four partial prior models that we tested above. Convergence was observed with 10 independent GA runs using each subset prior table by tracing the number of recovered edges according to the number of GA generations (left panel of Figure 2C), the fitness score according to the number of edges (right panel of Figure 2C), and the fitness score according to the number of the GA generations (Supplementary Figure S4B). Overall, the TF prior model covering distal binding and long-range interaction appeared to play a major part, while using only the eQTL prior data did not outperform the null prior model, replicating the subnetwork-scale prior evaluation results. Most importantly, incorporating DREs in network modeling was critical in recovering functional regulatory interactions with increased specificity and sensitivity. Additionally, we constructed and evaluated a leukemia network in the same manner as we did for the breast cancer network, and were able to confirm our findings on the importance of long-range TF prior modeling are not specific to one type of cancer (Supplementary Figure S5).

### Clinical evaluation of the global network

**Figure 3.**
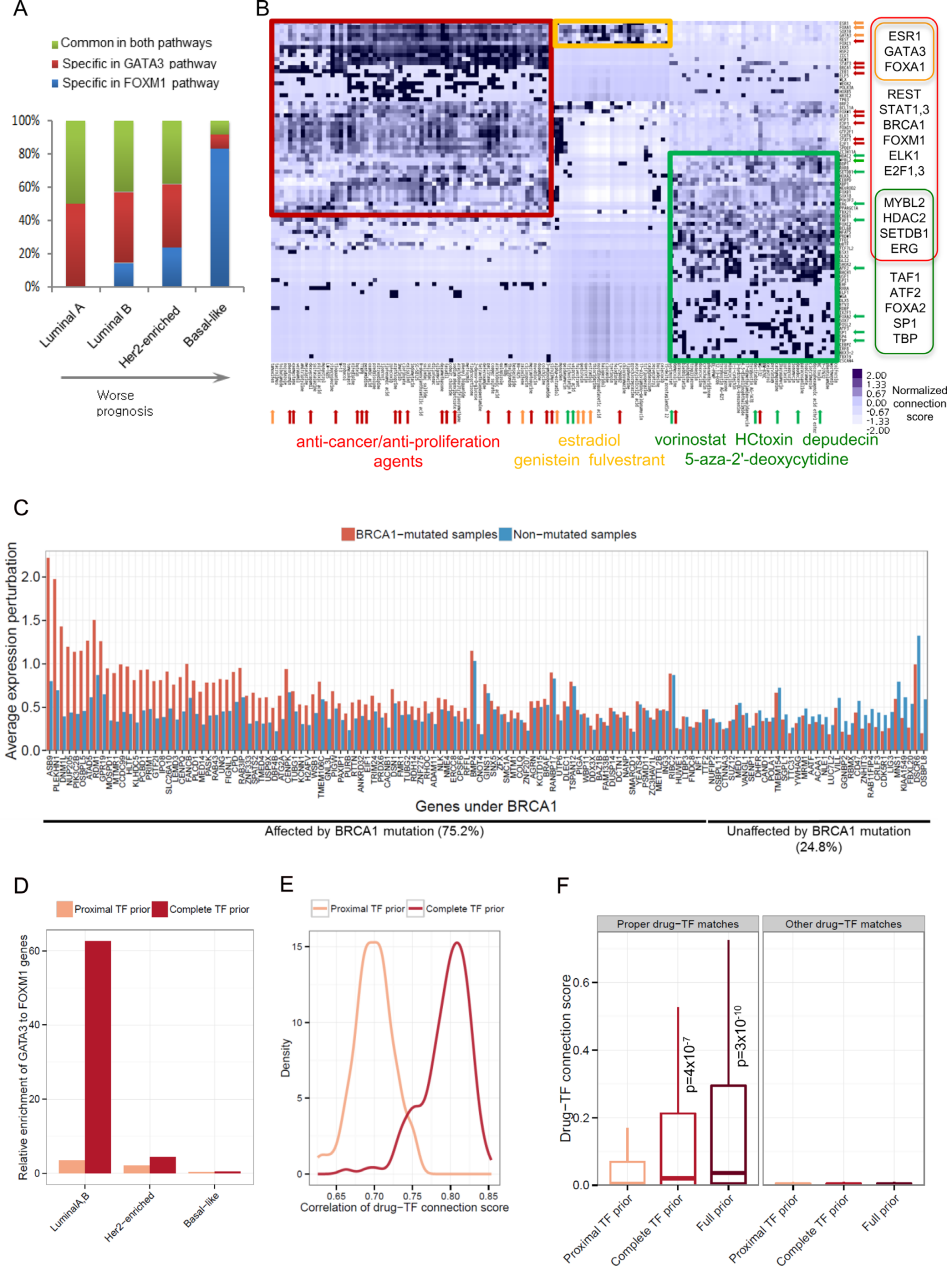
Tumor subclass and drug response analysis based on the global transcription network. (**A**) Frequency of subclass-specific genes present in the GATA3 or FOXM1 pathways. (**B**) Connectivity between small molecules (columns) and responsible TFs (rows) computed based on the mapping of transcription response signatures to the network. Unsupervised clustering was performed using the normalized connection scores, leading to three major clusters, a anti-cancer cluster (red), an epigenetic-drugs cluster (green) and an estrogen-receptor cluster (orange). (**C**) Expression perturbation of gene under BRCA1 in the global transcription network according to the mutation status of BRCA1 (**D**) Relative frequency of subclass-specific genes present in the GATA3 pathway as a ratio to the FOXM1 pathway in the two test networks based on the complete TF priors or proximal TF priors. (**E**) Correlations of the drug-TF connections scores between the full-scale full-prior network and the two test networks based on the complete or proximal TF priors. (**F**) Comparison of the connection scores for the selected proper drug-TF pairs (red, orange and green arrows in B) between the full-scale full-prior network and the two test networks based on the complete or proximal TF priors.

We next performed clinical evaluations. Breast cancer can be classified based on the status of three receptor proteins as luminal A, luminal B, HER2-enriched, or basal-like. The genes that were differentially expressed between tumor and normal tissue in a specific subclass were mapped to our transcription network to identify upstream regulators. GATA3, FOXA1 and FOXM1 were identified as key subclass regulators (Supplementary Figure S7), in agreement with previous findings based on annotated pathways (24). Because FOXA1 is a direct descendant of GATA3 in the network, we only used GATA3 as a representative regulator. The percentage of the subclass-specific descendants of GATA3 or FOXM1 was quantitatively correlated with the expected prognosis of the four subtypes (Figure 3A). For example, the highest percentage of the basal-like genes was specifically related to FOXM1, highlighting the role of this regulator in contributing to the aggressive nature of this subtype.

Next, we attempted a network-based interpretation of drug-response signatures. It has been difficult to understand the mechanism of action of drugs with only resulting gene expression signatures. Our causal network enabled the identification of the causal regulator that initiated the transcriptional changes in response to a given drug. In a previous work (39), MCF7 cells were treated with 155 small molecules, and gene expression changes were monitored. For each drug, we determined upstream transcription regulators. For each of these regulators, a connectivity score was calculated for each drug as the fraction of the responsive genes connected to the given regulator. This process yielded a matrix of connection scores representing how responsible each TF was for the transcriptional response to each drug. An unsupervised clustering identified three major clusters of drugs and biologically plausible TFs (Figure 3B). Estrogen and its receptor agonists/antagonists were associated with ESR1 and its associated factors, GATA3 and FOXA1 (44) (orange cluster). These luminal cancer regulators (7,24), along with other cancer-related TFs, were also responsive to anti-cancer agents (red cluster). Epigenetic drugs were linked to chromatin-modifying regulators (green cluster).

The drug response profile described above was based on luminal-type cells (MCF7). In a recent study (45), the EGFR inhibitor erlotinib was demonstrated to sensitize basal-like cancer cells (BT-20). We identified the genes responsive to erlotinib in BT-20 cells. There was a slight tendency toward specific up- and down-regulation of genes in the GATA3 and FOXM1 pathways, respectively (Supplementary Figure S8A). However, the largest fraction of genes was commonly present in the pathways of both GATA3 and FOXM1. We examined the relative distance of these genes to the two key regulators based on the number of intervening nodes in the network. The network distance of the up-regulated genes to GATA3 was shorter than to FOXM1 compared with the down-regulated genes (Supplementary Figures S8B and S9), supporting the hypothesis that this drug treatment may sensitize basal-like cells by inducing a luminal gene expression phenotype (45).

We also sought to interpret the clinical implications of BRCA1 mutations (46–48). Mutations will perturb the expression of the downstream genes in the regulatory network. The expression perturbation of the BRCA1-downstream genes was compared between patients with and without BRCA1 mutations. For each gene, the average expression perturbation of each group was obtained. As a result, the average expression perturbation of the BRCA1-mutated group was larger than that of the non-mutated group for 75.2% of the BRCA1-downstream genes (Figure 3C). This implies that BRCA1 mutations actually influence the transcription of genes that our network predicts as regulatory targets, and that clinical features related to BRCA1 mutations can be explained by action mechanisms of these target genes. Furthermore, 94% of these genes were responsive to putative BRCA1-targeting drugs as defined to have a positive drug connectivity score for BRCA1 in our drug response matrix in Figure 3B. Only 5% of genes that were not influenced by BRCA1 mutations were responsive to the BRCA1-targeting drugs. Therefore, the predicted BRCA1-targeting drugs may indeed interact with the BRCA1 protein. These findings illustrate clinical and therapeutic implications of our network construction and analysis schemes.

### Biological implications of distal regulation in the global network

To assess the biological importance of incorporating DRE priors in network modeling, we ran pilot MCMC with 10 seed networks as described above by using either the TF priors covering both distal and proximal regulation or the TF priors considering proximal binding only. We evaluated these MCMC global networks by referring to the curated functional interaction database as we did for subnetwork evaluation in Figure 2A. As a result, we found that DRE information enhances the precision of the global network (Supplementary Figure S6A). Also, we computed the relative fraction of potentially true-positive links as the F1 score for each node by querying the full-scale GA-MCMC network. Higher F1 scores were achieved when using DRE information (Supplementary Figure S6B).

We then tested the performance of the two test networks in terms of their capability to explain breast cancer subclasses and drug response. The specific regulation of luminal A/B subclass genes by GATA3 relative to FOXM1 as we observed in the full-scale network (Figure 3A) was pronounced much better by the complete TF priors than the proximal TF priors (Figure 3D). The drug-TF connectivity scores obtained by analyzing the full network (Figure 3B) were compared with the scores obtained from the two test networks in the same manner. Markedly higher correlations of the connectivity scores were observed when the complete TF priors were used (Figure 3E). We then selected properly matched drug-TF pairs (the red, green, orange arrows in Figure 3B from the anti-cancer cluster, epigenetic-drugs cluster and estrogen-receptor cluster, respectively). While the connectivity scores of the proper matches were much higher than those of the other matches overall, the complete-TF test network produced significantly higher scores than the proximal-TF test network (Figure 3F), indicating that modeling distal regulation is critical in understanding gene expression programs underlying drug response.

We next sought to estimate the functional effects of DRE and PRE TF binding based on the degree of enrichment of priors in the functional network (prior recovery rate). We first classified each prior relationship into two prior types (TF prior or eQTL prior) or four binding types (TF binding to PRE, TF binding to DRE tethered to PRE, TF binding to PRE tethered to PRE, or TF binding to DRE tethered to PRE tethered to PRE). The fraction of the prior relationships recovered in the functional network (i.e. the number of relationships in the functional network divided by the number of relationships in the prior list) was obtained for each class. This fraction was divided by the expected degree of enrichment to obtain an odds ratio. To estimate expected enrichment, we obtained all possible pairs of genes that were used for functional network construction and counted the fraction of the gene pairs that were present in the final functional network as an edge without regard to the regulatory sign. The final odds ratio was used as the enrichment score for each class of prior relationships.

**Figure 4.**
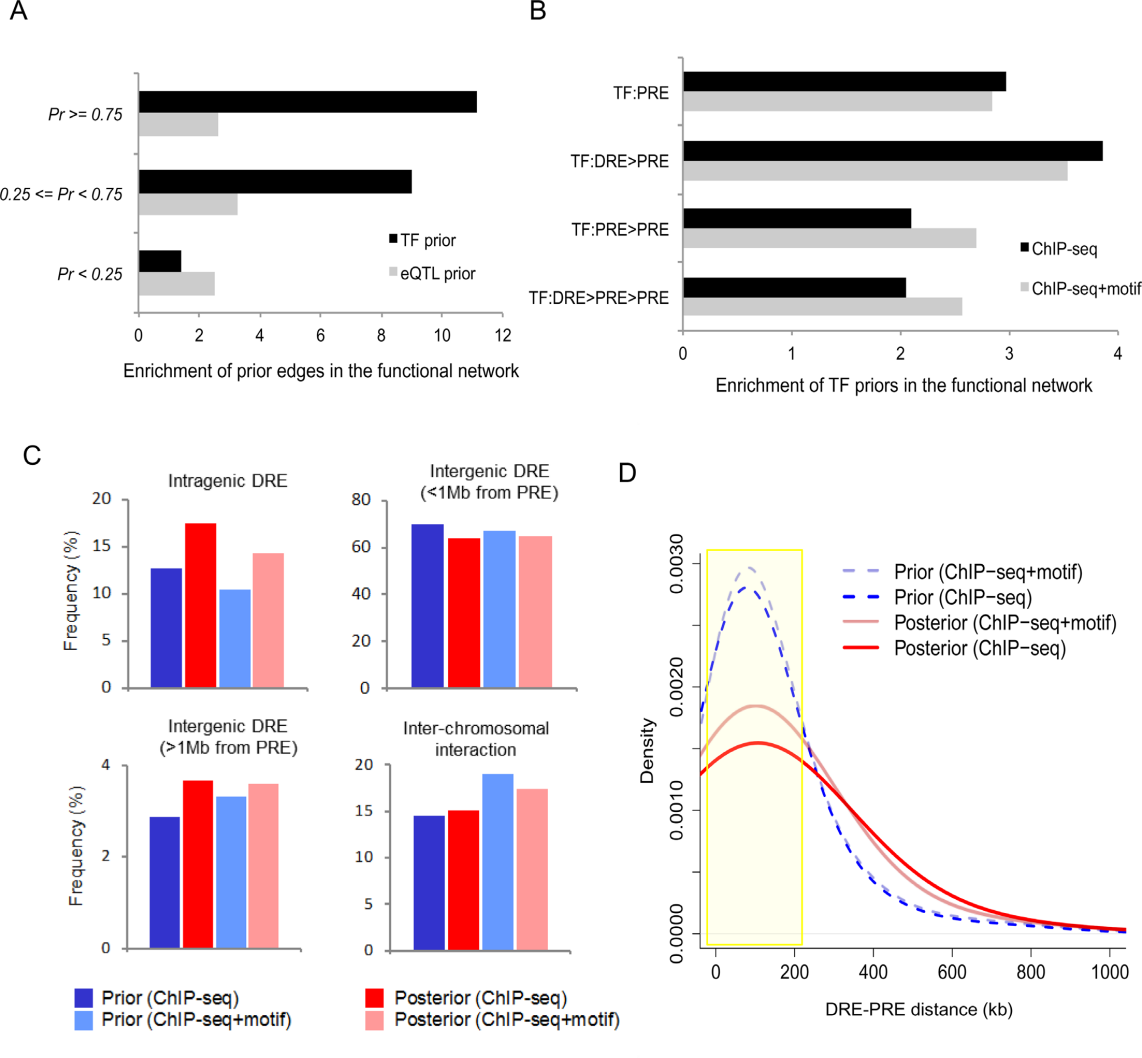
Characterization of distal regulatory interactions in the prior framework and functional network. (**A**) Recovery rate of the TF prior and eQTL prior interactions in the functional network according to their prior probability. (**B**) Recovery rate of the TF prior interactions in the functional network according to regulator binding mode. (**C**) Frequency of DRE–PRE interactions in the prior (physical) and posterior (functional) network according to their positioning. (**D**) Frequency of DRE–PRE distances in the prior (physical) and posterior (functional) network.

As shown in Figure 4A, the high-probability (Pr ≥ 0.75) TF priors were >10 times more likely than expected to be retained in the posterior network, with enrichment gradually decreasing in proportion to the prior probability (black bars). This pattern was not observed with the eQTL prior model (gray bars). A high recovery rate for edges and nodes was observed when TFs bound DREs that were tethered to PREs (Figure 4B and Supplementary Figure S10). Regulator-target functional associations via promoter-promoter interactions were relatively low, although they were higher than expected (Figure 4B). The majority of the prior and posterior DRE–PRE interactions were intra-chromosomal and involved either intragenic or short-range (<1 Mb) intergenic DREs (Figure 4C). The posterior fraction of the intragenic DREs was higher than the prior fraction (Figure 4C). Intriguingly, a distant DRE–PRE pair was more likely to be functional than closely spaced DREs and PREs (Figure 4D). The distance distribution suggests that physical chromatin contacts are frequently formed between DREs and PREs that are <200 kb apart, but a sizable fraction of them may be non-functional; in contrast, functional interactions tend to span longer distances (Figure 4D). These results underscored the functional importance of the long-range actions of DREs.

### Network analysis of transcriptional drivers and risk genes

We then characterized the transcriptional target genes of breast cancer-risk SNPs identified in multiple GWASs (27). Most (>80%) of these transcriptional risk genes were connected to functional DREs. These genes may increase cancer susceptibility when their transcription is genetically misgregulated. Our primary question centered on the role of these risk genes, particularly in relation to transcriptional cancer drivers. Here we considered two types of transcriptional cancer drivers. Coding driver factors were defined as TFs containing recurrent missense mutations in their coding region (24–26). A total of 29 coding driver factors were identified. Regulatory driver factors were defined as TFs carrying in *trans* recurrent non-coding mutations in their binding sites at a high frequency. To identify such regulatory driver factors, we mapped non-coding motif-breaking mutations in 21 breast cancer patients (40,41) onto our gene network (Supplementary Figure S11), discovered recurrently mutated genes and examined responsible TFs for these genes. Most (∼80%) of these mutations were concentrated in functional DREs. Based on *in silico* and clinical simulation (see Methods), we identified 17 regulatory driver factors as having a significantly large number of recurrently affected target genes (*P* < 0.05).

**Figure 5.**
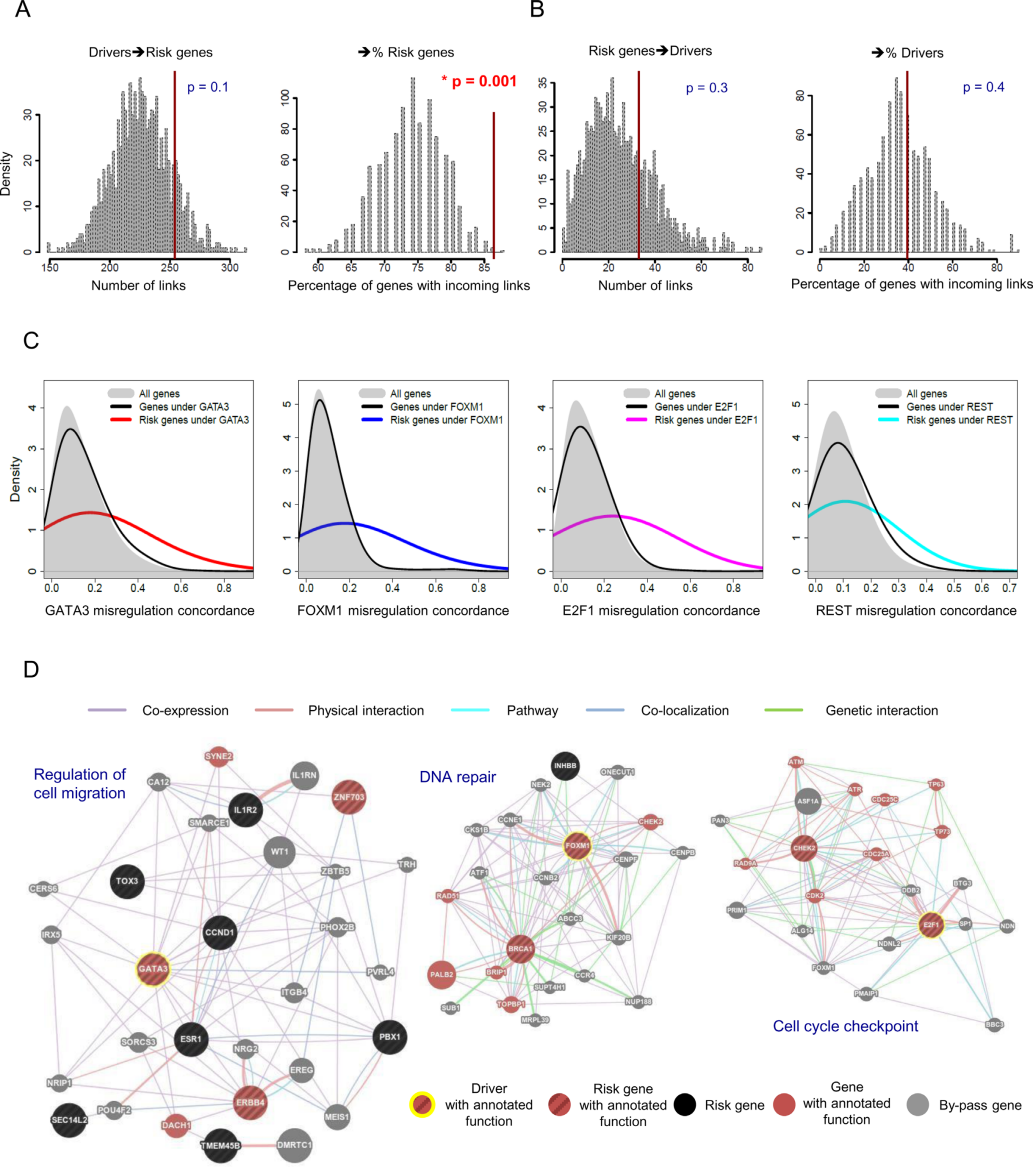
Functional connectivity between the transcriptional drivers and risk genes. (**A**) The statistical significance of the number of causal links directed from the driver to risk genes (left) and that of the percentage of the risk genes with incoming links (right) were determined based on 1000 random samplings of the same number of genes as the true risk genes. (**B**) The same statistical tests were performed for the number of causal links directed from risk to driver genes (left) and the percentage of the driver genes with incoming links (right). (**C**) Misregulation concordance between selected key transcriptional drivers (GATA3, FOXM1, E2F1 and REST from left to right) and all genes in the network (gray), downstream genes in the network (black) and downstream genes that are risk genes (red, blue, pink and cyan). (**D**) GeneMANIA functional association analysis of transcriptional drivers (GATA3, FOXM1 and E2F1 from left to right) and their downstream risk genes with high misregulation concordance. Gene nodes connected via co-expression, physical interaction, pathway, co-localization, and genetic interaction and shared enriched functions (*q* < 0.1) are displayed.

We investigated our TF prior-based network connections as well as interrogated the breast cancer eQTL data (23) and other eQTL maps to identify a total of 90 risk genes (Supplementary Figure S11). We identified a subnetwork consisting of these risk genes and the transcriptional cancer drivers (the coding or regulatory driver factors as described above), and then examined the structure of this subnetwork. The number of driver-to-risk connections (left panel of Figure 5A) was >7-fold higher than that of risk-to-driver connections (left panel of Figure 5B). This result is due in part to the intrinsic high outdegrees of the drivers because large numbers of driver-to-risk connections were generally observed when sampling the risk genes while fixing the drivers (left panel of Figure 5A; a marginal *P*-value of 0.1). However, the percentage of the risk genes with incoming links from the drivers (>85%) was rarely observed in the permutation (*P* = 0.001; right panel of Figure 5A). These findings imply that the transcriptional risk genes are specifically regulated directly or indirectly by the transcriptional cancer drivers.

We sought to examine whether the tumorigenic changes in the drivers could induce the misregulation of the risk genes. To this end, we obtained the expression data for the tumor-normal matched samples from the TCGA data portal and examined the concordance between the differential expression status of key transcriptional cancer drivers and that of all genes, all downstream genes and downstream risk genes. As illustrated by the colored curves in Figure 5C and Supplementary Figure S12A, downstream risk genes tended to be selectively misregulated in patients with driver alterations. GATA3 is frequently mutated in luminal subtypes (24). GATA3 coding mutations also appeared to selectively induce the misregulation of downstream risk genes (Supplementary Figure S12B).

These data suggest that risk genes might participate in somatic tumor development initiated by their upstream driver regulators. To better understand the transcriptional role of risk genes in transformation and cancer progression, we examined their cellular function in the context of the pathway of their upstream drivers. For a few representative driver regulators, the downstream risk genes that exhibited high misregulation concordance were identified, and their shared function was investigated. A functional cluster of GATA3 and its nine cooperating downstream risk genes, including ERBB4 and ZNF703, was identified as having a shared function of cell migration regulation (leftmost of Figure 5D). Similarly, BRCA1 and CHEK2 were suggested to participate in cancer development by modulating DNA repair and cell cycle checkpoints in collaboration with FOXM1 and E2F1, respectively (middle and right of Figure 5D).

### Role of DNA variants in the cooperation of drivers and risk genes

We observed that only a subset of the downstream genes responded to tumorigenic changes in the drivers. The concordance of misregulation between the drivers and their downstream genes was not higher than that between the drivers and irrelevant genes (black curves versus gray shades in Figure 5C). Only the risk genes were responsive to the alteration of their upstream drivers (colored curves in Figure 5C). We suspected that DNA variation might play a synergistic role in mediating the selective susceptibility of the risk genes to the drivers. In other words, cancer risk variants might increase the transcriptional sensitivity of the target genes to driver alteration, for example, by modulating the DNA binding affinity of the drivers themselves, the co-binding regulators of the drivers, or the downstream regulators of the drivers.

**Figure 6.**
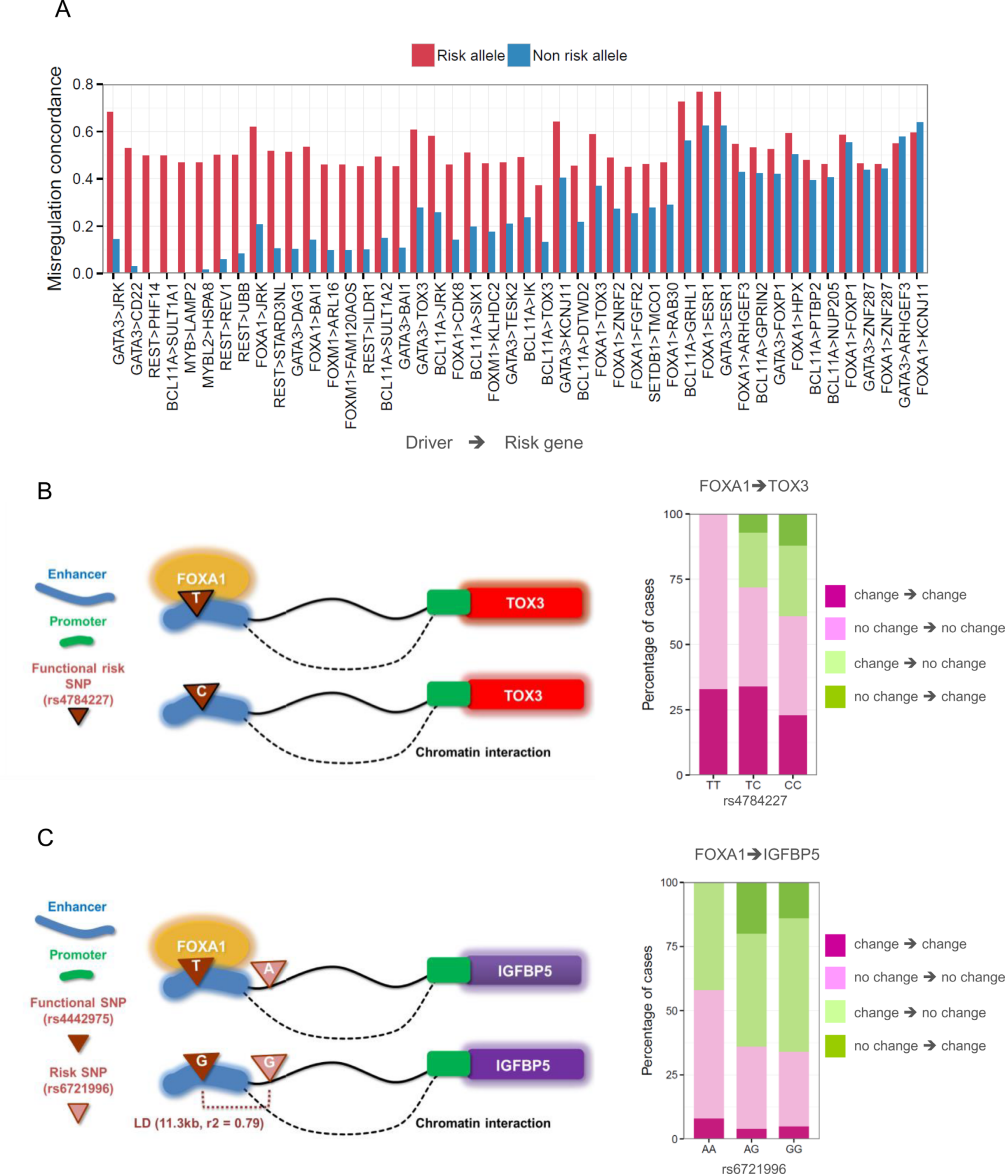
DNA variant-mediated transcriptional cooperation of cancer drivers with risk genes. (**A**) The patients the carry the previously reported risk alleles present higher misregulation concordance between the associated risk genes and their upstream drivers than those carrying the non-risk alleles. Shown are the cases in which the risk group exhibits significant concordance (*P* < 0.01). (**B** and **C**) Examples of the synergistic role of risk SNPs supported by experimental evidence. A higher fraction of concordant cases (dark and light pink) and a lower fraction of discordant cases (dark and light green) are observed with the risk allele than with the non-risk allele. (B) Higher concordance for the risk allele T than for the non-risk allele C at rs4784227 suggests that in patients carrying the risk allele, FOXA1 binding affinity increases and TOX3 misregulation is more specifically associated with the expression change of FOXA1 (49). (C) Higher concordance for the risk allele A at rs6721996 suggests that in patients carrying the risk allele, FOXA1 binding to the functional site harboring rs4442975 increases and IGFBP expression is more responsive to FOXA1 expression changes (50).

To test a potential synergistic role for risk alleles, we obtained genotype data for the tumor-normal TCGA samples and inferred risk alleles by comparing allele frequencies in the TCGA patients with those in normal genomes (42). For driver-risk pairs for which genotype data are available, we computed misregulation concordance within the risk group of patients and within the non-risk group of patients separately. We first identified the cases in which misregulation concordance was significant (*P* < 0.01) in either the risk group or the non-risk group. The risk group exhibited higher concordance in 86% of the identified cases (Figure 6A). For example, the risk allele (T) of rs4784227 increased the susceptibility of TOX3 expression to tumorigenic FOXA1 alterations (Figure 6B). This risk allele increases the binding affinity of FOXA1 to the SNP-containing DRE of TOX3 (49). In another example, the risk allele (A) of rs6721996 increased the susceptibility of IGFBP5 transcription to aberrant FOXA1 expression in tumor (Figure 6C). This risk allele was in linkage disequilibrium with the T allele of rs4442975, which has been shown to increase the binding affinity of FOXA1 to a DRE connected to IGFBP5 (50).

Above, we provided examples in which the risk allele directly disrupted the binding site of the driver regulator. However, oncogenic driving changes may propagate down the regulatory network through multiple downstream regulators. Many risk factors might increase the transcriptional susceptibility of their associated genes to oncogenic network perturbation by altering the transcription of one of the multiple upstream regulators. This demonstrates why a global regulatory network is required to fully understand the mechanisms underlying cancer development and susceptibility. We were not able to apply the same test for mutations because no whole-genome mutation data accompanied by tumor-normal gene expression profiles are available. However, the findings with risk SNPs imply that the impact of mutations may be well pronounced not independently but in synergy with driver alteration.

## DISCUSSION

Current approaches to regulatory network reconstruction in humans heavily or solely depend on gene expression patterns without a systematic modeling of physical and functional regulator-target relationships, thereby limiting our understanding of the mechanism of action of regulatory variations at the systems level. Furthermore, global network reconstruction has been required to understand how genetic perturbation in regulatory elements can be translated into systems perturbation via the propagation of the primary transcriptional changes through the gene transcription network.

In the present study, we constructed a global gene regulatory network at an unprecedented level of biological coverage and accuracy based on an efficient computational algorithm and precise modeling of genomic regulatory interactions. In most cases, TF binding, especially in distal regulatory regions, was likely to play a causal role in target gene regulation. However, to our surprise, causal relationships inferred based on genetic association were not well supported by actual gene expression patterns. It is not clear at present whether this phenomenon is specific to the expression and eQTL data sets used in this work or whether the assumed causality between cis- and trans-associated genes does not properly reflect the true biological causality.

The functional importance of the long-range actions of enhancers stood out, thereby underscoring the need to incorporate the chromatin interactome into regulatory networks and to assess their functional validity on the basis of the expression patterns of the physical target genes. Intriguingly, a fraction of physical chromatin contacts between closely spaced DREs and PREs appeared to be non-functional. This may reflect non-specific contacts between a promoter and multiple adjacent DREs or may indicate chromatin loopings poised for transient gene induction only in specific conditions (51). It has been difficult to understand the mechanism of action of drugs with only gene expression signatures. Our network approach proved successful in identifying responsible regulators that govern transcriptional changes in response to particular drugs. We were also able to delineate gene expression programs underlying different patient subtypes, and to demonstrate how this can lead to novel personalized therapeutics when combined with our network-based drug response analysis.

We performed a systematic identification of the genes affected by non-coding regulatory variations. Distal regulatory regions were enriched for such variations, again highlighting the functional importance of long-range chromatin interactions. Remarkably, our findings reveal functional connectivity between genetic factors that increase cancer susceptibility and somatic events that drive oncogenesis. This link was unexpected because risk genes are usually thought to confer susceptibility to cancer rather than to directly participate in tumorigenesis driven by somatic events. Our results suggest that risk genes might contribute to transformation and cancer progression via a molecular mechanism similar to that via which they increase cancer risk. Risk factors are polymorphic and thus can potentially affect the whole body, suggesting that single risk genes may not be able to exert individual phenotypic effects strong enough to drive oncogenesis. However, tumorigenic changes in the drivers may lead to concerted changes in multiple risk genes. Therefore, a tumor can develop via the combined effects of the misregulation of multiple risk genes, while the selective misregulation of these genes can be enhanced by the synergistic action of DNA variation. Genetic risk factors at cancer susceptibility loci may increase the risk of cancer onset by rendering their target genes more susceptible to oncogenic, coding or regulatory, somatic changes in cancer-driving factors. These illustrate how the network-based systematic elucidation of genetic perturbation can contribute to a better understanding of regulatory mechanisms underlying a wide range of biological processes.

## SUPPLEMENTARY DATA

Supplementary Data are available at NAR Online.

SUPPLEMENTARY DATA
