# Supplementary material for: Global transcription network incorporating distal regulator binding reveals selective cooperation of cancer drivers and risk genes
Source: Nucleic Acids Res. 2015 May 22;43(12):5716–29. doi: 10.1093/nar/gkv532 (PMC4499150; doi:10.1093/nar/gkv532)
Supplement: SUPPLEMENTARY DATA [file supp_43_12_5716__index.html]

Global transcription network incorporating distal regulator binding reveals selective cooperation of cancer drivers and risk genes — SUPPLEMENTARY DATA 

# Global transcription network incorporating distal regulator binding reveals selective cooperation of cancer drivers and risk genes

## SUPPLEMENTARY DATA

- SUPPLEMENTARY DATA
- SUPPLEMENTARY DATA
