# Supplementary material for: Global transcription network incorporating distal regulator binding reveals selective cooperation of cancer drivers and risk genes
Source: Nucleic Acids Res. 2015 May 22;43(12):5716–29. doi: 10.1093/nar/gkv532 (PMC4499150; doi:10.1093/nar/gkv532)
Supplement: SUPPLEMENTARY DATA [file supp_gkv532_nar-00304-n-2015-File003.pptx]

## Slide 1
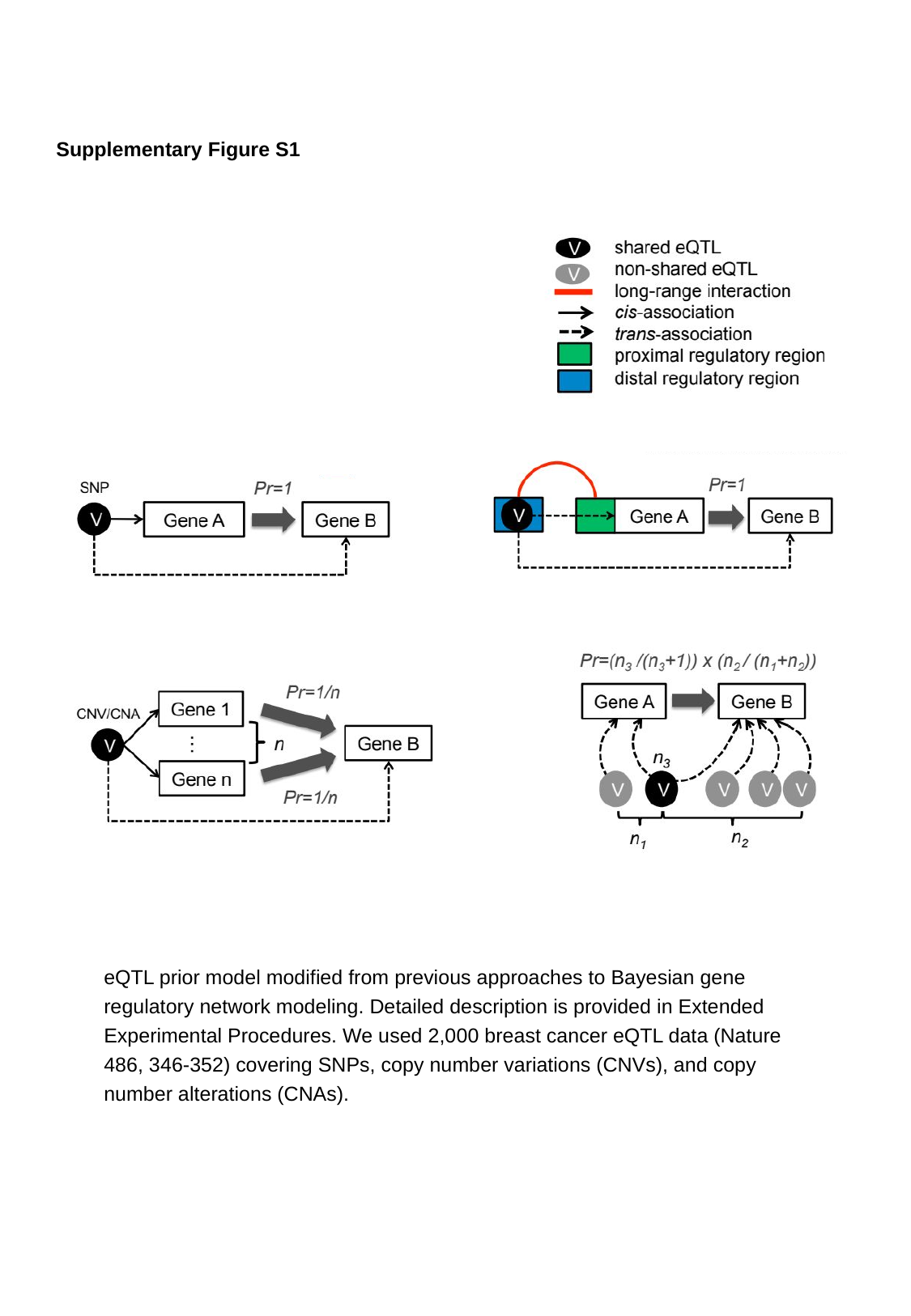

Supplementary Figure S1
eQTL prior model modified from previous approaches to Bayesian gene regulatory network modeling. Detailed description is provided in Extended Experimental Procedures. We used 2,000 breast cancer eQTL data (Nature 486, 346-352) covering SNPs, copy number variations (CNVs), and copy number alterations (CNAs).

## Slide 2
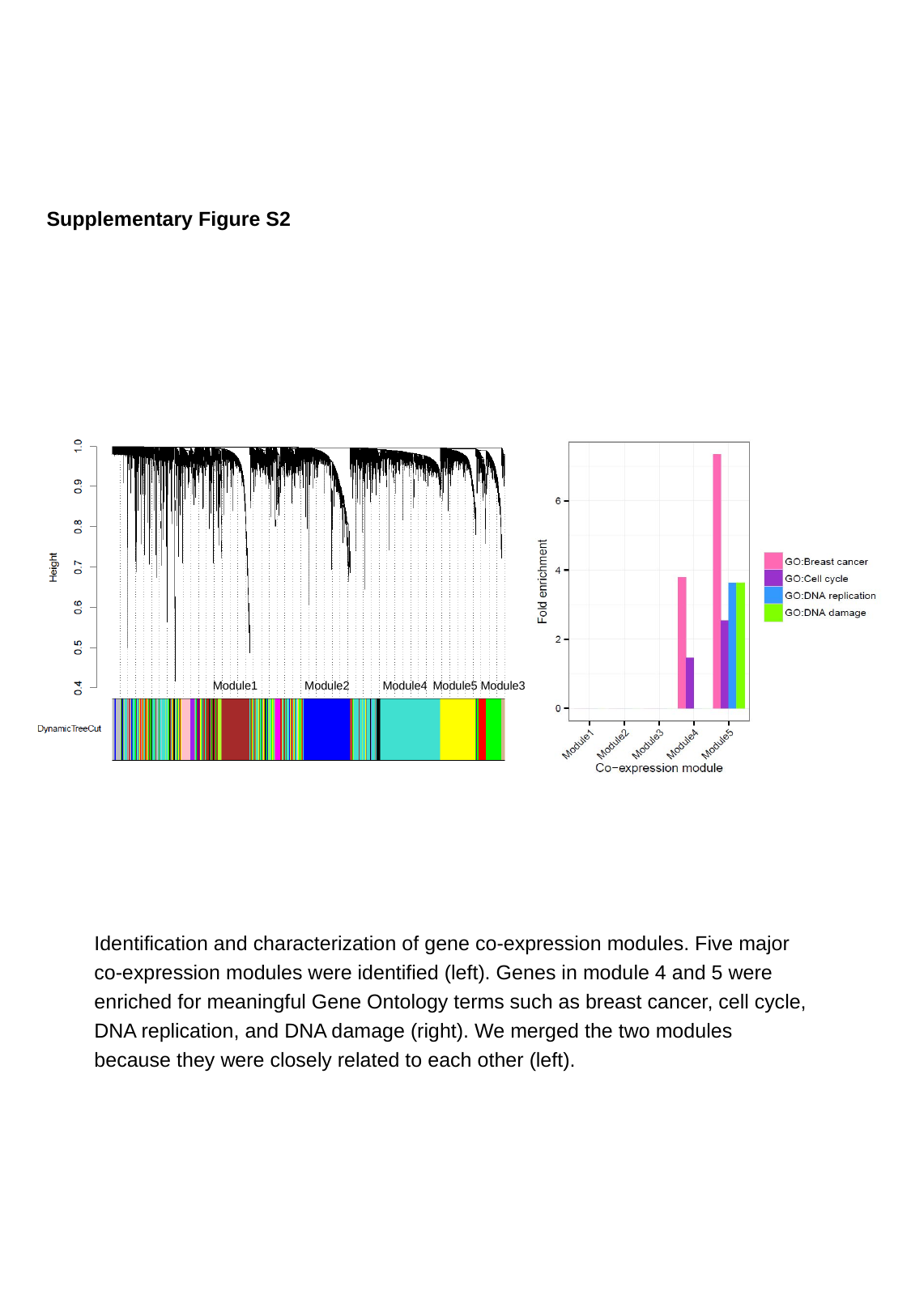

Supplementary Figure S2
Module1
Module2
Module4
Module5
Module3
Identification and characterization of gene co-expression modules. Five major co-expression modules were identified (left). Genes in module 4 and 5 were enriched for meaningful Gene Ontology terms such as breast cancer, cell cycle, DNA replication, and DNA damage (right). We merged the two modules because they were closely related to each other (left).

## Slide 3
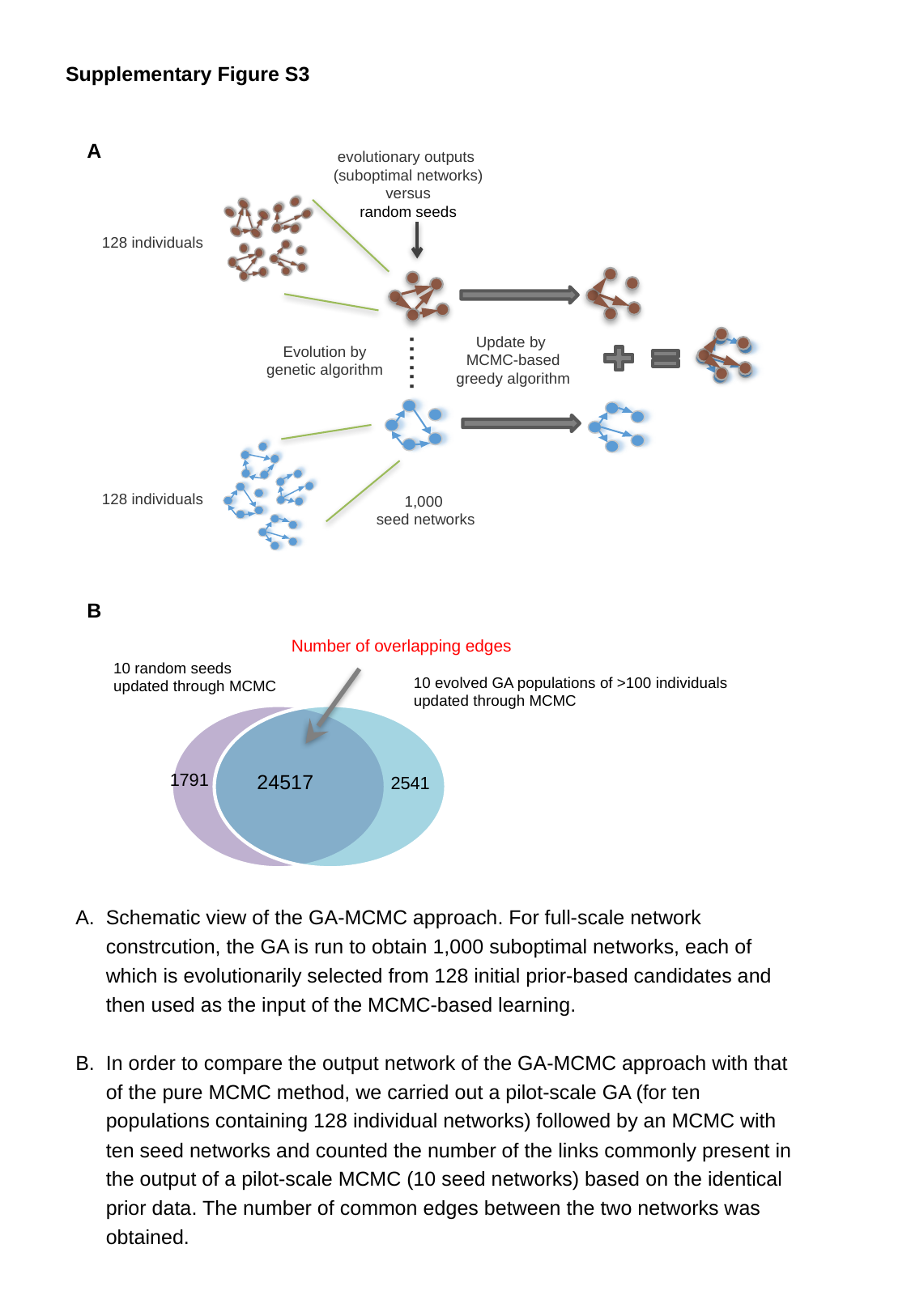

128 individuals
128 individuals
Evolution by
genetic algorithm
evolutionary outputs
(suboptimal networks)
versus
random seeds
......
1,000
seed networks
Update by
MCMC-based
greedy algorithm
Supplementary Figure S3
A
B
Number of overlapping edges
10 random seeds
updated through MCMC
10 evolved GA populations of >100 individuals
updated through MCMC
1791
24517
2541
Schematic view of the GA-MCMC approach. For full-scale network constrcution, the GA is run to obtain 1,000 suboptimal networks, each of which is evolutionarily selected from 128 initial prior-based candidates and then used as the input of the MCMC-based learning.
In order to compare the output network of the GA-MCMC approach with that of the pure MCMC method, we carried out a pilot-scale GA (for ten populations containing 128 individual networks) followed by an MCMC with ten seed networks and counted the number of the links commonly present in the output of a pilot-scale MCMC (10 seed networks) based on the identical prior data. The number of common edges between the two networks was obtained.

## Slide 4
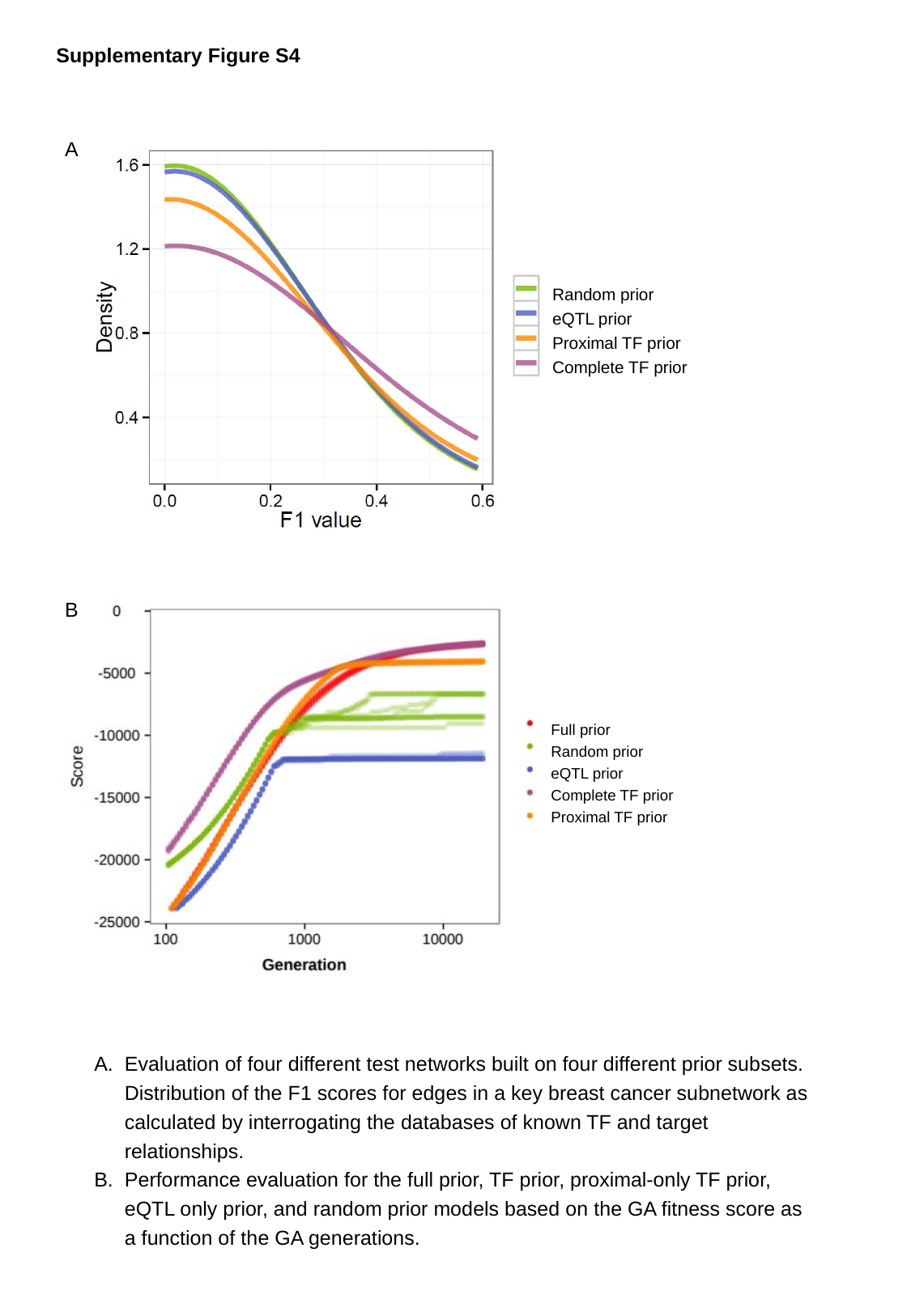

Supplementary Figure S4
A
Random prior
eQTL prior
Proximal TF prior
Complete TF prior
B
Full prior
Random prior
eQTL prior
Complete TF prior
Proximal TF prior
Evaluation of four different test networks built on four different prior subsets. Distribution of the F1 scores for edges in a key breast cancer subnetwork as calculated by interrogating the databases of known TF and target relationships.
Performance evaluation for the full prior, TF prior, proximal-only TF prior, eQTL only prior, and random prior models based on the GA fitness score as a function of the GA generations.

## Slide 5
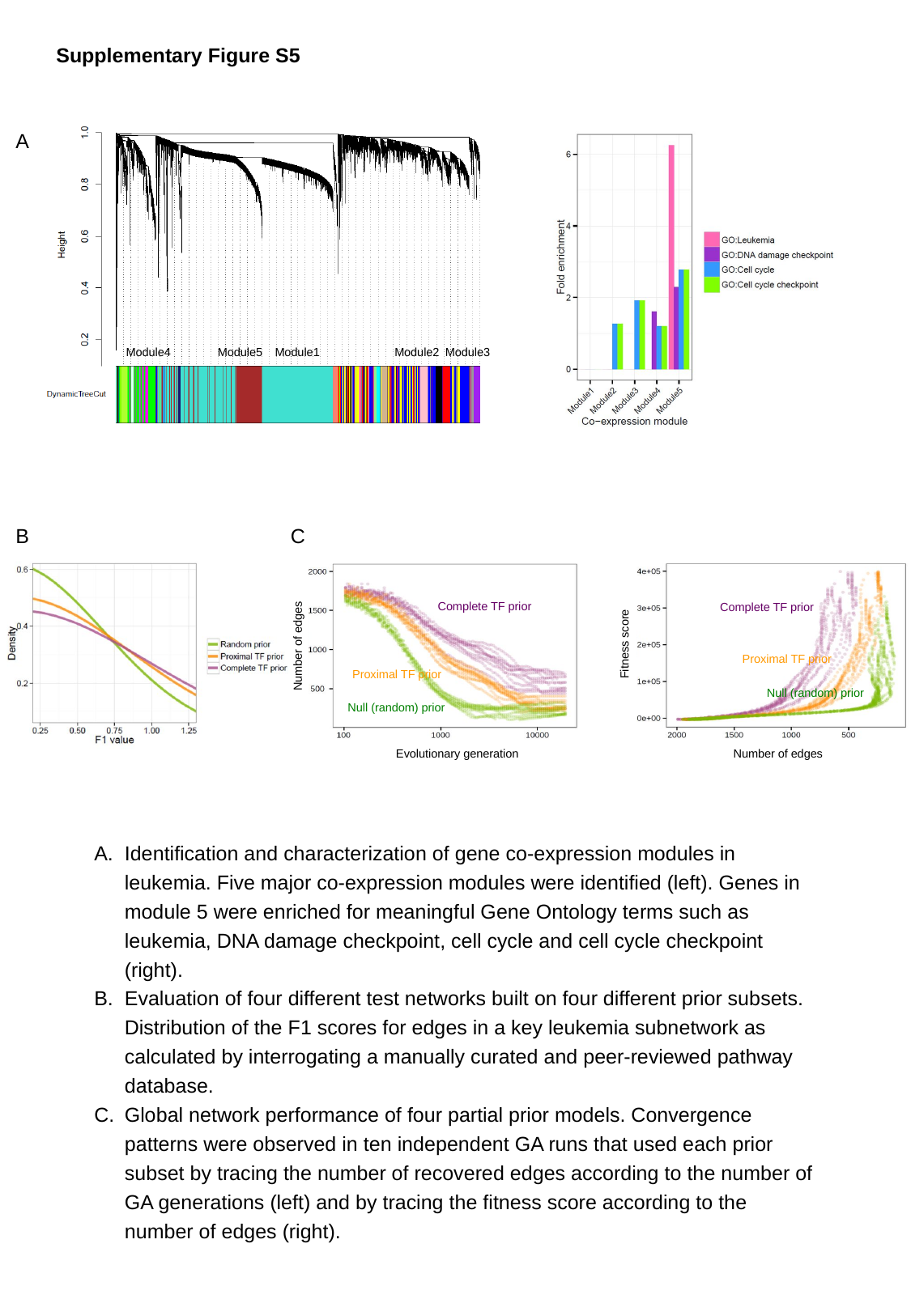

Supplementary Figure S5
A
Module4
Module5
Module1
Module2
Module3
B
C
Complete TF prior
Proximal TF prior
Null (random) prior
Fitness score
Number of edges
Complete TF prior
Number of edges
Proximal TF prior
Null (random) prior
Evolutionary generation
Identification and characterization of gene co-expression modules in leukemia. Five major co-expression modules were identified (left). Genes in module 5 were enriched for meaningful Gene Ontology terms such as leukemia, DNA damage checkpoint, cell cycle and cell cycle checkpoint (right).
Evaluation of four different test networks built on four different prior subsets. Distribution of the F1 scores for edges in a key leukemia subnetwork as calculated by interrogating a manually curated and peer-reviewed pathway database.
Global network performance of four partial prior models. Convergence patterns were observed in ten independent GA runs that used each prior subset by tracing the number of recovered edges according to the number of GA generations (left) and by tracing the fitness score according to the number of edges (right).

## Slide 6
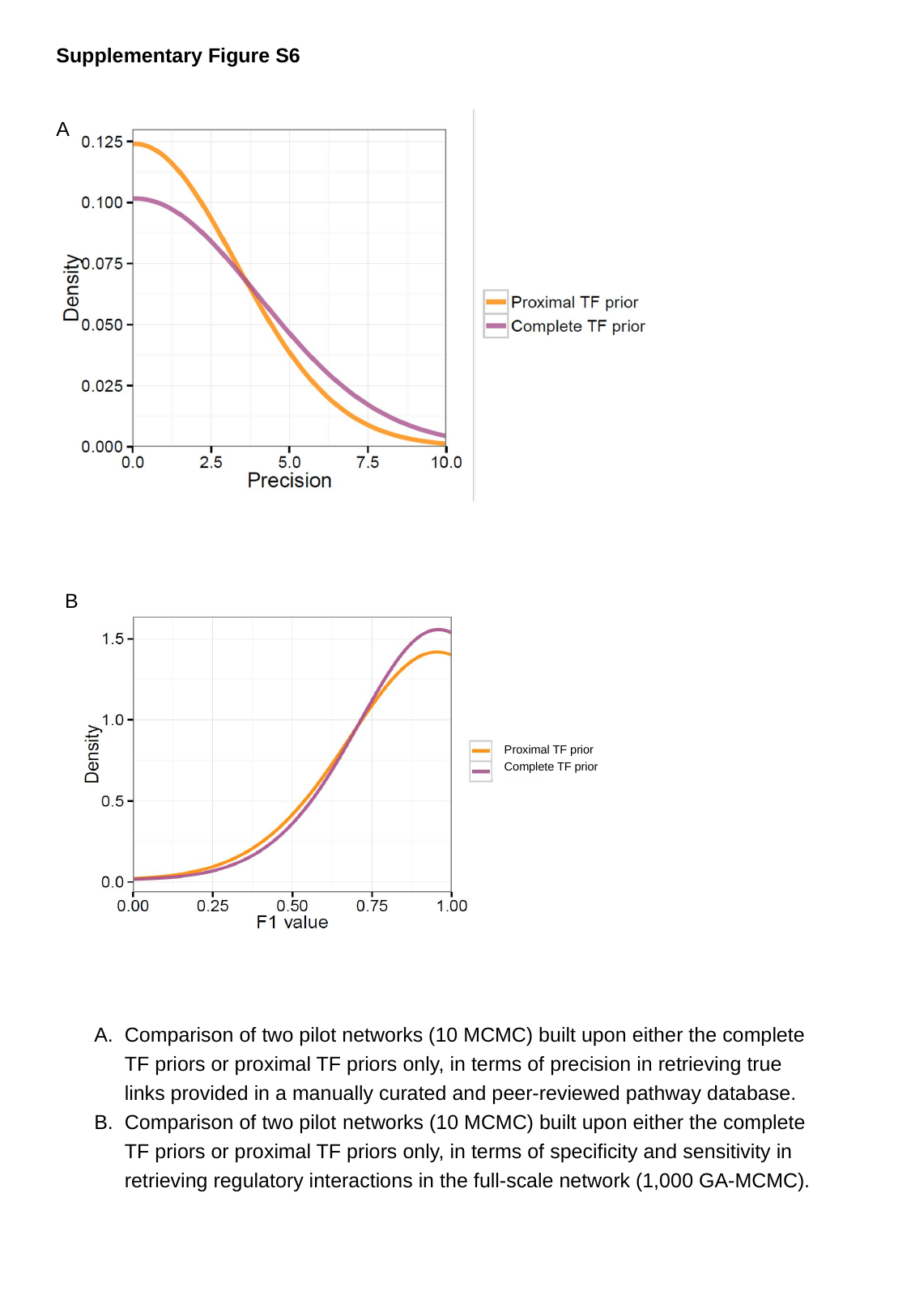

Supplementary Figure S6
A
B
Proximal TF prior
Complete TF prior
Comparison of two pilot networks (10 MCMC) built upon either the complete TF priors or proximal TF priors only, in terms of precision in retrieving true links provided in a manually curated and peer-reviewed pathway database.
Comparison of two pilot networks (10 MCMC) built upon either the complete TF priors or proximal TF priors only, in terms of specificity and sensitivity in retrieving regulatory interactions in the full-scale network (1,000 GA-MCMC).

## Slide 7
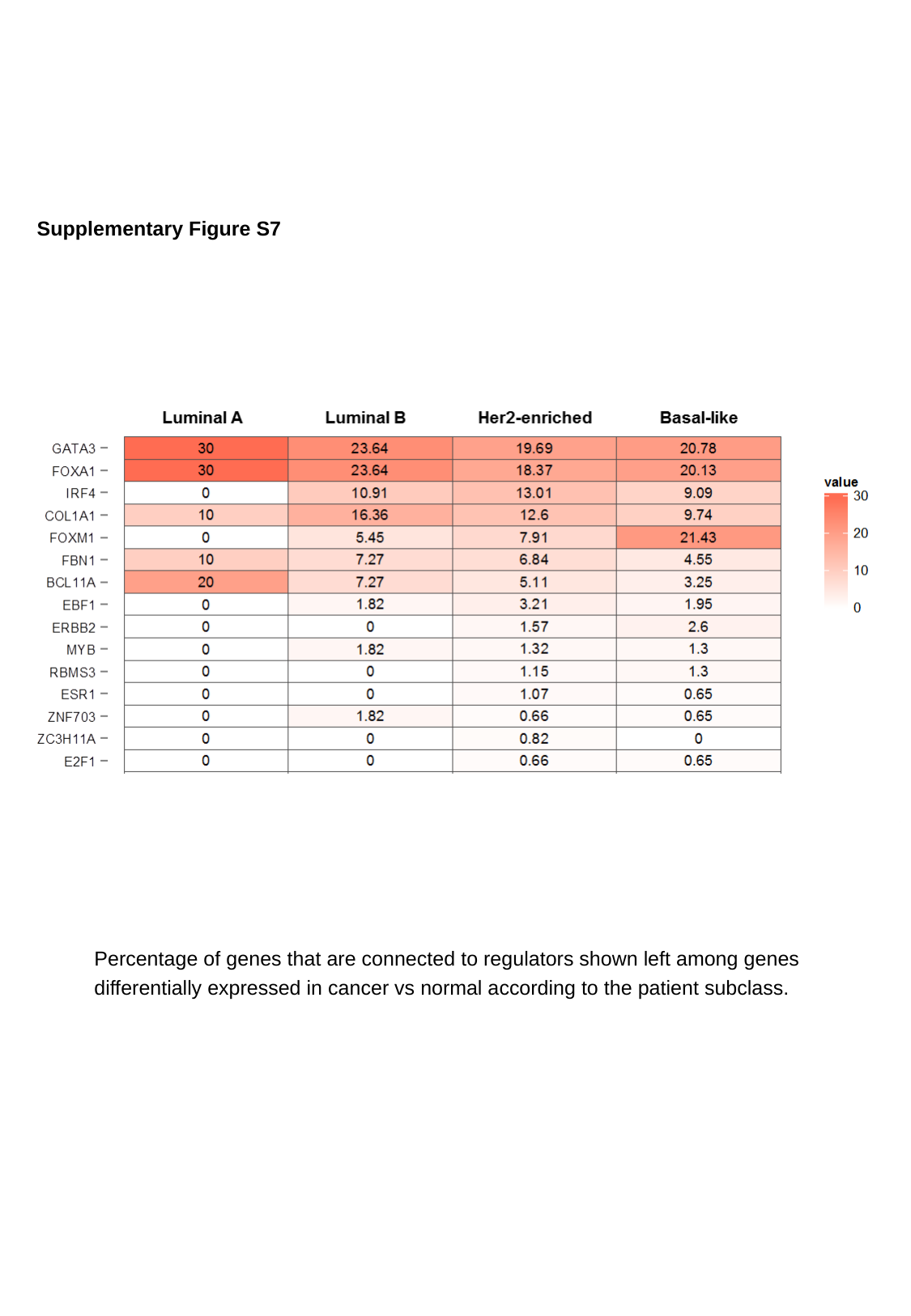

Supplementary Figure S7
Percentage of genes that are connected to regulators shown left among genes differentially expressed in cancer vs normal according to the patient subclass.

## Slide 8
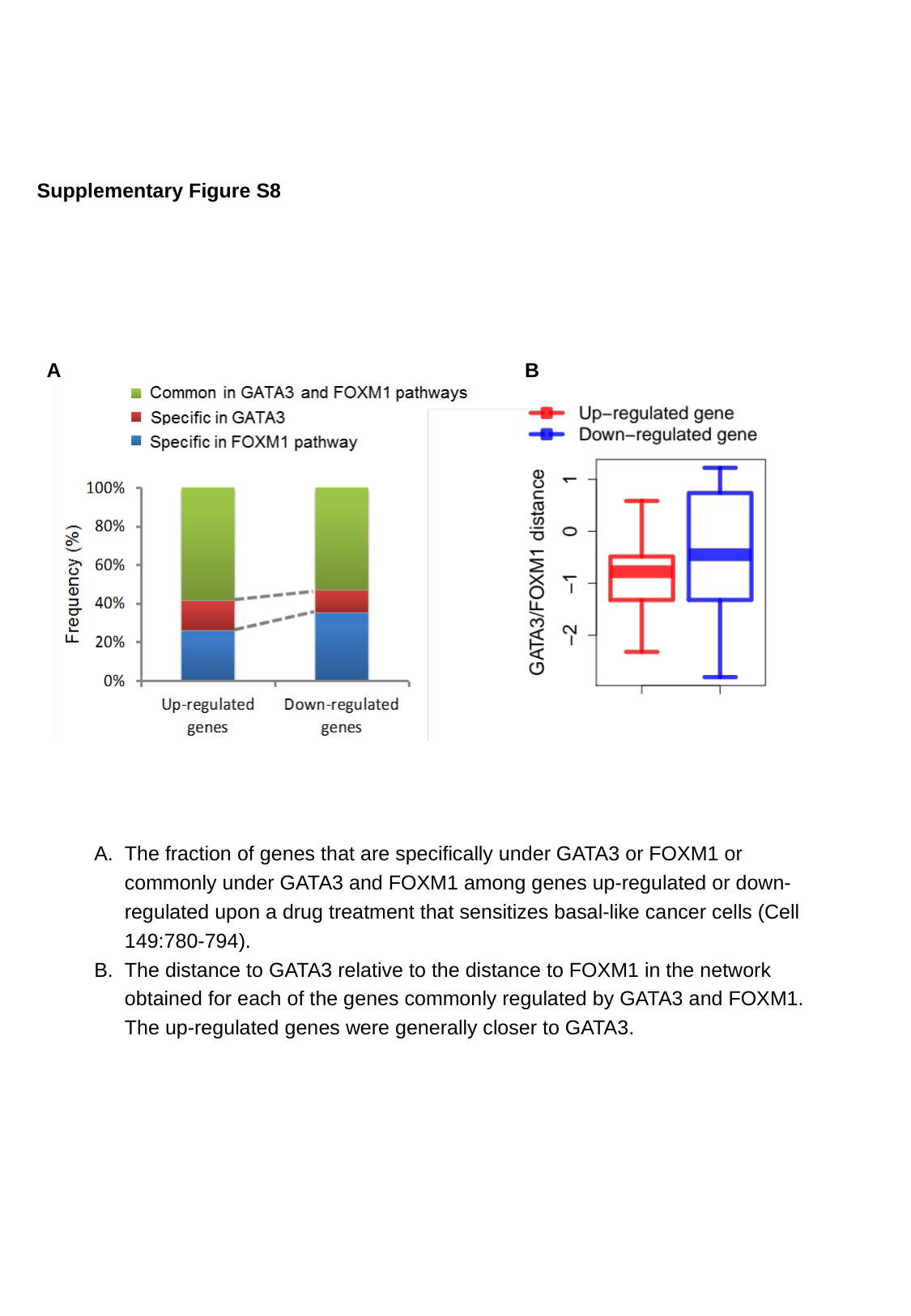

Supplementary Figure S8
A
B
The fraction of genes that are specifically under GATA3 or FOXM1 or commonly under GATA3 and FOXM1 among genes up-regulated or down-regulated upon a drug treatment that sensitizes basal-like cancer cells (Cell 149:780-794).
The distance to GATA3 relative to the distance to FOXM1 in the network obtained for each of the genes commonly regulated by GATA3 and FOXM1. The up-regulated genes were generally closer to GATA3.

## Slide 9
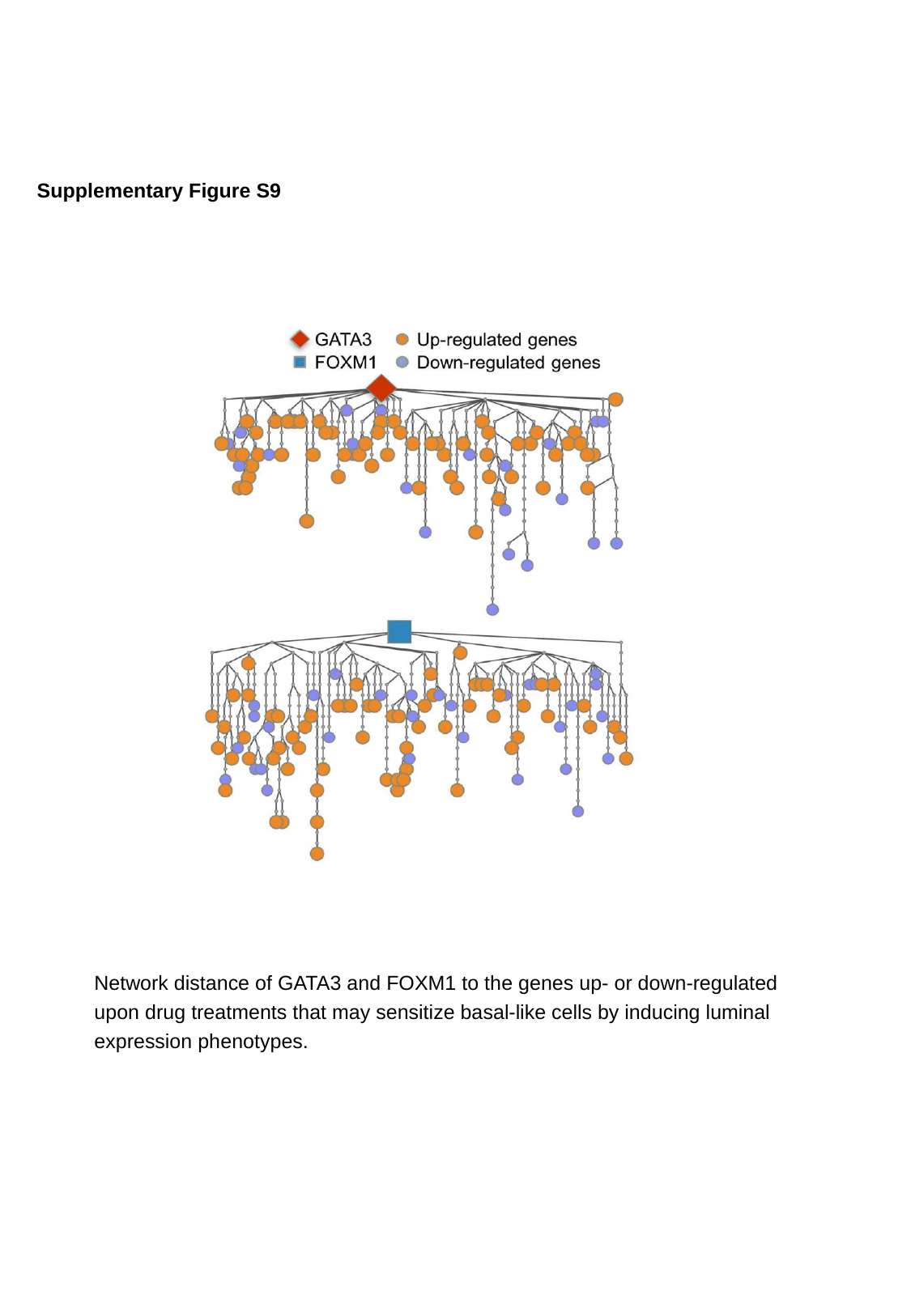

Supplementary Figure S9
Network distance of GATA3 and FOXM1 to the genes up- or down-regulated upon drug treatments that may sensitize basal-like cells by inducing luminal expression phenotypes.

## Slide 10
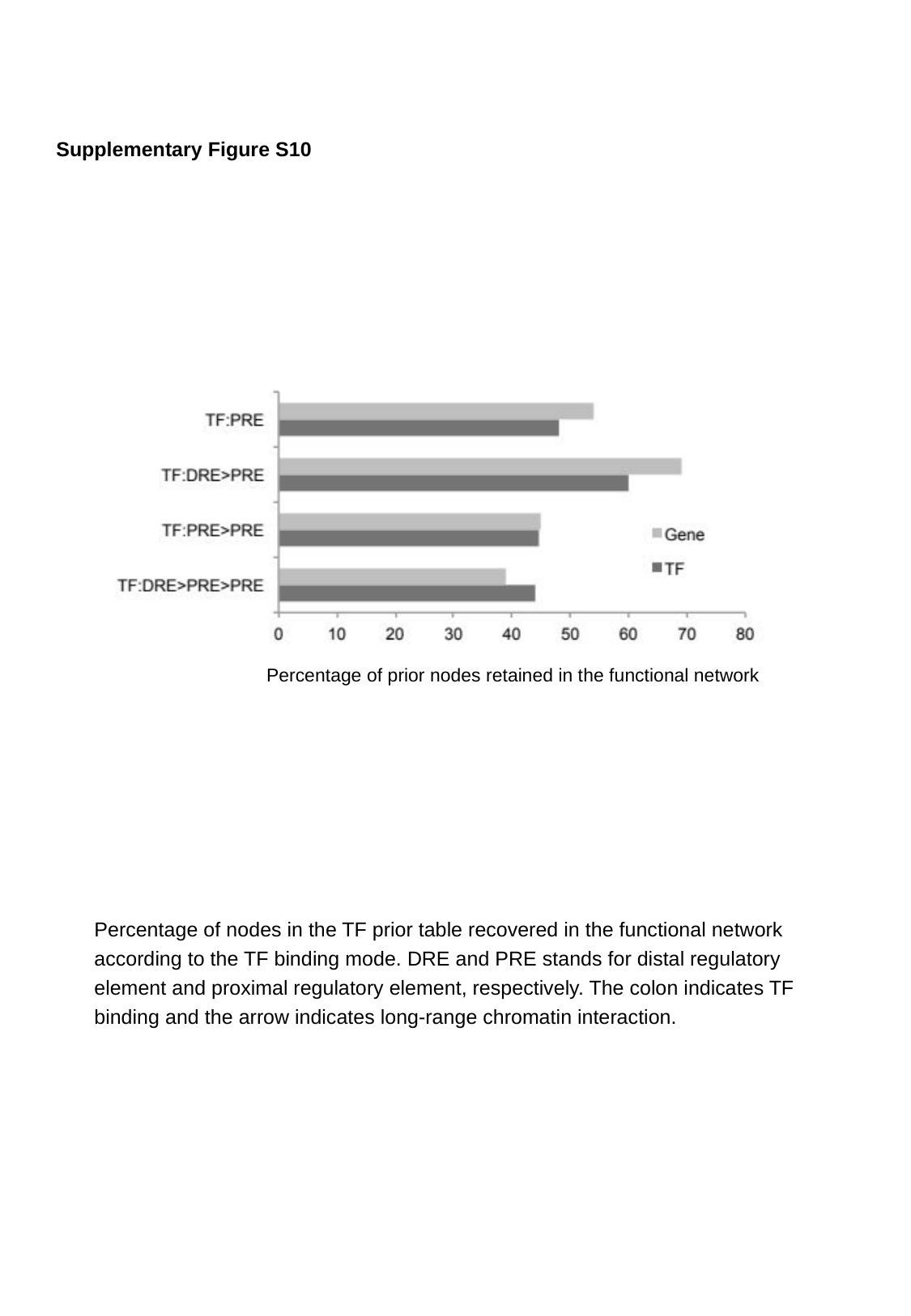

Supplementary Figure S10
Percentage of prior nodes retained in the functional network
Percentage of nodes in the TF prior table recovered in the functional network according to the TF binding mode. DRE and PRE stands for distal regulatory element and proximal regulatory element, respectively. The colon indicates TF binding and the arrow indicates long-range chromatin interaction.

## Slide 11
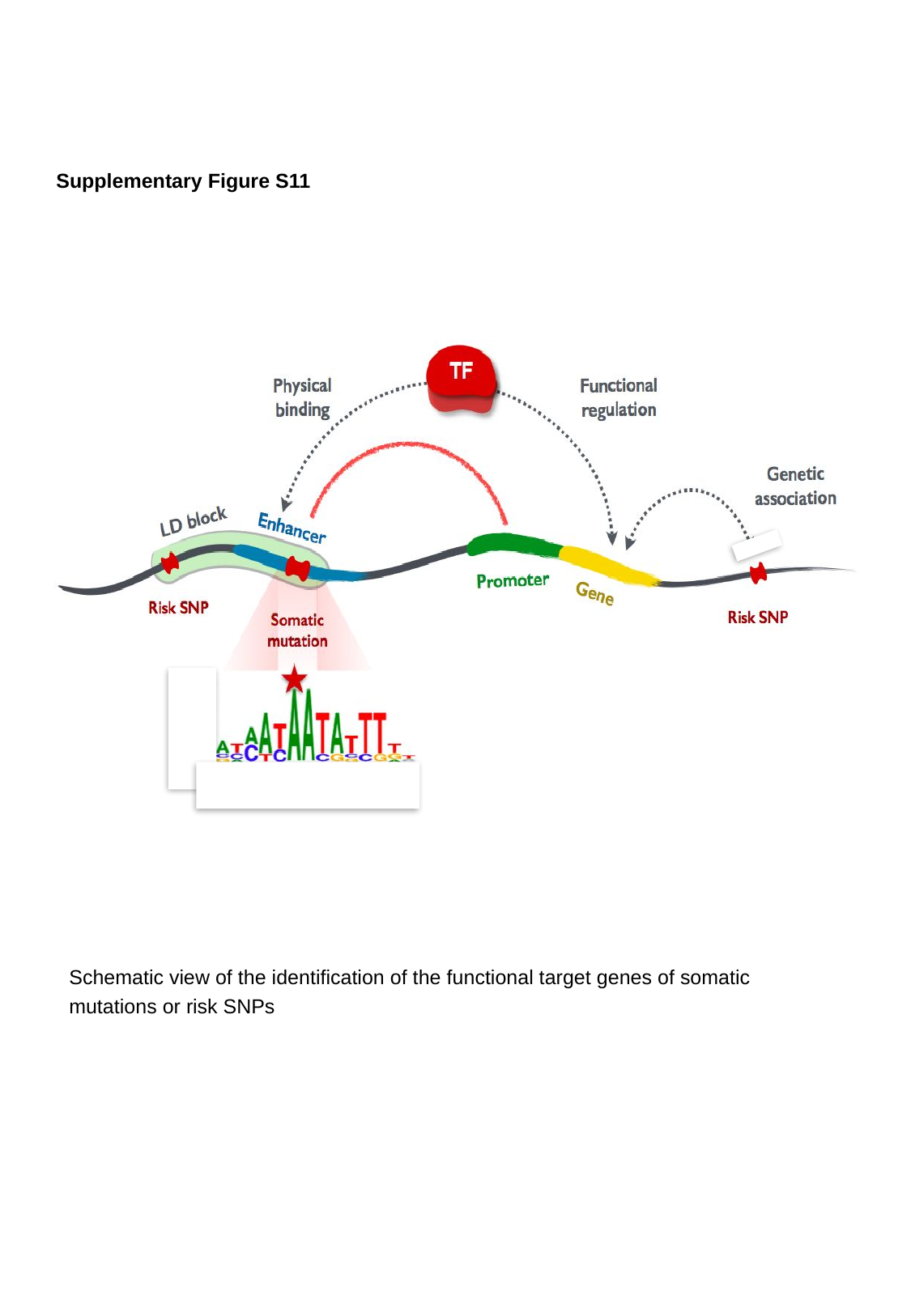

Supplementary Figure S11
Schematic view of the identification of the functional target genes of somatic mutations or risk SNPs

## Slide 12
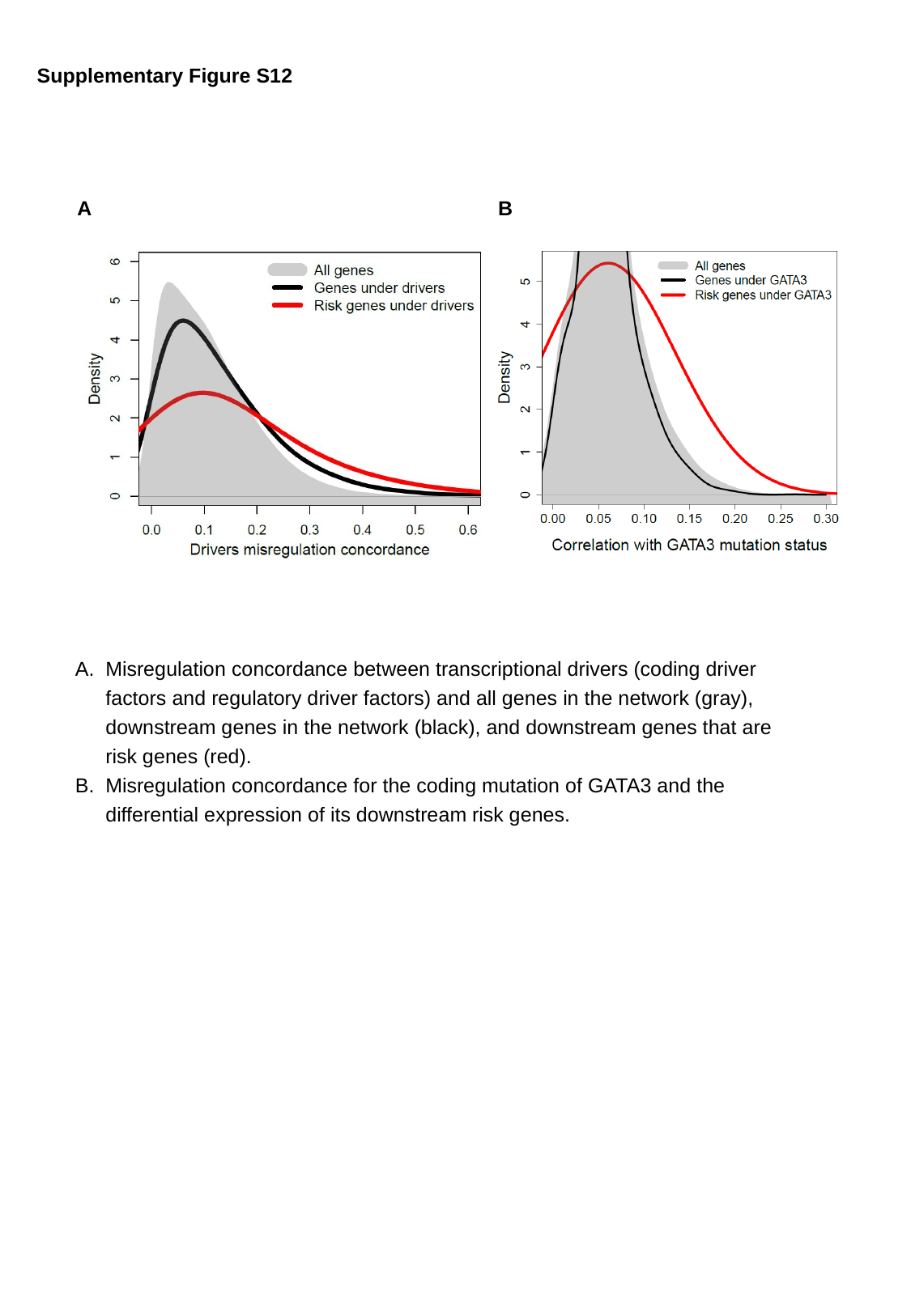

Supplementary Figure S12
A
B
Misregulation concordance between transcriptional drivers (coding driver factors and regulatory driver factors) and all genes in the network (gray), downstream genes in the network (black), and downstream genes that are risk genes (red).
Misregulation concordance for the coding mutation of GATA3 and the differential expression of its downstream risk genes.
