# Supplementary material for: Global transcription network incorporating distal regulator binding reveals selective cooperation of cancer drivers and risk genes
Source: Nucleic Acids Res. 2015 May 22;43(12):5716–29. doi: 10.1093/nar/gkv532 (PMC4499150; doi:10.1093/nar/gkv532)
Supplement: SUPPLEMENTARY DATA [file supp_gkv532_nar-00304-n-2015-File004.docx]

**Supplementary Methods**

**TF binding, TF motif, and chromatin interaction datasets**

Data for a total of 822 ChIP-seq experiments for 159 TFs in 104 different cell lines, including breast cancer cell lines MCF-7 and T47D, were obtained from the ENCODE project database (http://genome.ucsc.edu/ENCODE/). TF binding regions were defined based on the peak location of ChIP-seq tags as identified in each experiment by the ENCODE project groups. For additional TF binding information, we mapped the position weight matrix (PWM) of each TF available in the TransFac (1) 2013.1 release onto regulatory regions as defined by DHSs in the two breast cancer cell lines (MCF-7 and T47D) as obtained from the ENCODE database. For PWM mapping, we used the FIMO tool (2) of the MEME SUITE package (3) as previously suggested (4). A total of 378 TFs were mapped to regulatory regions (DHSs) in the breast cancer cell lines. One hundred seven TFs were only available in the ENCODE data but not in the TransFac database. When combined with the binding (ChIP-seq) data, binding sites for a total of 485 unique TFs were obtained and used to build our TF prior model.Chromatin interactions mediated by RNA polymerase II (Pol2) in MCF-7 cells as profiled by chromatin interaction analysis by paired-end tag (ChIA-PET) sequencing (5) were obtained from the UCSC ENCODE track and incorporated into our TF and eQTL prior probabilistic model. ESR1 binding and ESR1-mediated chromatin interaction in MCF-7 cells as profiled by ChIA-PET were also utilized (6). CTCF is also capable of providing chromatin contacts for enhancer-mediated transcription regulation (7). Therefore, a total of 75,861 CTCF-mediated chromatin interactions that were previously produced as part of the ENCODE project were added to our model.

**Breast cancer gene expression and genetic association datasets**

In a previous work (8), a total of 1,992 primary breast tumours, consisting of a discovery set of 997 samples and a validation set of 995 samples were profiled for gene expression, single nucleotide polymorphism (SNP), copy number variation (CNV), and somatic copy number alteration (CNA). We employed the data of association mapping between gene expression level and each genetic marker (i.e., either SNP, CNV, or CNA). The identified cis-associations (<3Mb between the transcript and the genetic marker) and trans-associations were used to build our eQTL prior model. For our Bayesian learning of causal network structure, the microarray and RNA-seq data of The Cancer Genome Atlas (TCGA) encompassing 1,386 tumour samples (536 on Agilent G450A microarray and 850 on Illumina Hi-Seq) were obtained (htpp://www.cancergenome.nih.gov). Expression data for the matched normal samples (62 pairs on Agilent G450A microarray and 108 pairs on Illumina Hi-Seq) were also obtained from the TCGA data portal.

**Construction of the TF prior model**

For direct PRE binding, the edge from the TF gene to the transcript gene was assigned a prior probability of 1 (Fig. 1B). We also used TF ChIP-seq data in cell types other than breast cancer by mapping their peak locations to breast cancer DHSs. For the binding of multiple, m, TFs in the other cell types, a prior probability of 1/m was assigned to each link from the m TF genes to the transcript gene (Fig. 1C). For multiple, n, promoters via promoter-promoter contacts, a prior probability of 1/n was assigned to each of the n links directed from the TF gene to the n transcripts (Fig. 1B). Again, the number of candidate TFs, m, was considered for non-breast cancer binding such that a prior probability of 1/n/m was assigned to each of the links connecting the m TF genes to the n putative target transcripts (Fig. 1C).

The most important advantage of our prior model is that the physical target genes of DREs can be identified based on experimental evidence of long-range chromatin interactions. For distal regulator binding with experimental evidence of chromatin looping, the edge connecting the TF gene to the transcript gene was assigned a prior probability of 1 when the binding event was supported by ChIP-seq or within-DHS motifs in breast cancer cells (Fig. 1D). We also used TF ChIP-seq data in non-breast-cancer cells with reduced prior probabilities (Fig. 1E). When a TF-bound DRE was connected to a PRE, which was then linked to multiple, n, PREs, a prior probability of 1/n or 1/m/n was assigned to each of the n links from the TF gene to the n transcripts (Fig. 1D and 1E).

**Construction of the eQTL prior model**

Genetic association of gene expression performed by expression QTL (eQTL) mapping can be used to help infer regulatory networks because directionality for the causality of transcription regulation can be provided unlike evidence obtained solely from gene expression patterns. Because genes associated with cis-acting eQTLs directly reflect the source of perturbation, they tend to appear in the top layers of the Bayesian networks (9). Therefore, these genes can be assumed to be a causal regulator of the genes associated in trans with the same genetic marker (10–14, 9). Following the previous approaches to Bayesian regulatory network construction, the cis-acting eQTL gene was defined as a parent of trans-acting eQTL gene with a prior probability of 1 for a shared genetic marker (10, 12). We employed a previous eQTL dataset that used SNPs, CNVs, and CNAs for their genetic marker across 1992 primary breast tumours (8). When the genetic marker is not represented as a SNP but as a CNV or CNA, there can be multiple (n) genes in the duplicated or deleted block and one of these candidates will be the cis-acting eQTL gene, or the causal regulator of the downstream, trans-acting eQTL genes. Therefore, a prior probability of 1/n is assigned to the edge between the n cis-associated genes and trans-associated genes. One of the advantages of our eQTL prior model is that long-range chromatin interactions are included to enable a more detailed modeling. Certain genetic associations might have been interpreted as trans-acting because the marker is distant (>3Mb) from the associated gene although there is direct chromatin looping between the marker and the gene, in which case this association should be labeled cis-association. Therefore, a probability of 1 was assigned to the connection between long-distance-acting eQTL genes and purely trans-acting eQTL genes for the shared variants. Again, a prior probability of 1/n was assigned when there is ambiguity among n candidate genes. When a common locus was associated in trans with two genes and there was no experimental evidence for physical chromatin contact between the marker and the genes, directionality for the causality of regulation was inferred based on the number of QTLs associated with each gene as follows. Suppose Gene A and Gene B has n1 and n2 unique associations, respectively, while sharing n3 QTLs in common. It can be posited that genes in the top layers of the causal network will be influenced by a smaller number of genetic factors (i.e., less frequently perturbed); in other words, when n1<n2, it can be assumed that Gene A is upstream of Gene B. In this case, the prior probability of the causal direction (Gene A -> Gene B) will be proportional to the number of Gene B QTLs (i.e., n2) as compared to the number of Gene A QTL (i.e., n1). The probability of the connection itself without regard to directionality will be proportional to the number of the shared loci (i.e., n3). Taken together, we assign a prior probability of (n3/(n3+1)) x (n2/(n1+n2)) when n1<n2. This rule has been employed with modification from a previous study (12). In this manner, a total of 373,672 prior relationships between 9,561 putative regulators and 9,504 putative targets were modeled as summarized in Fig. S1.

**Integration of the prior data and gene expression data**

The eQTL and TF prior relationships were combined. For the duplicate pairs of regulators and targets, the maximum prior probability was chosen. Finally, a total of 3,328,575 prior relationships between 9,679 putative regulators and 13,026 putative target genes were used as the input of our Bayesian learning. Among 485 TFs that were present in either ChIP-seq or motif dataset, 436 were associated with DHSs in the breast cancer cell lines and also were covered by the TCGA breast cancer gene expression data and thus were included in the final prior input. For our Bayesian learning of causal network structure, the TCGA gene expression data encompassing 1,386 tumour samples were used. Continuous expression levels of each gene were partitioned into discrete groups indicating high, medium, and low expression. We observed that the partitioning by the k-means clustering was not as consistent as the outcome of the Partitioning Around Medoids (PAM) method, which is known to be more robust to noise than the k-means clustering. Therefore, we ran PAM for each gene by using the fpc R package. The partitioning was performed for the expression matrix of the 536 Agilent G450A samples and that of the 850 Illumina Hi-Seq samples separately. A total of 14,930 genes were commonly present in both platforms. By combining the expression dataset with the merged prior table, we were left with 13,047 genes including 436 TFs with physical binding information. Other TFs without binding information were also included in our Bayesian learning procedure.

**Network data representation for GA**

The logical representation of a chromosome is a two-dimensional matrix of *N* by *N* where *N* is total number of genes in the network. Each element of array may have a value -1, 0, or 1. There is one-to-one correspondence between all possible network structures and all possible representations of a chromosome. Because the chromosome matrix is very sparse, in our GA operation, each chromosome was physical implemented as a set of one-dimensional arrays of linked lists, in order to reduce memory space allocated for each chromosome and to increase the computation performance of crossover and evaluation.

**GA network initialization**

The previous MCMC-based network learning for a subset of genes used 1,000 random seeds (10–14, 9). Because *N*(N-1)* possible links should be tested during learning for *N* nodes, the increase of the number of genes from a subnetwork scale (e.g., 2,000) to a global scale (e.g., 20,000) will require at least 100-fold more initial seeds for the search of optimal networks. In this regard, our GA started with 1,000 initial populations containing 128 chromosomes, which is in effect equivalent to 128,000 random seeds for the MCMC-based greedy algorithm. An initial population was created prior to evolution based mainly on prior information such that the frequency of each link in the initial population was proportional to the prior probability assigned to the link. The frequency of a link with a prior probability of α in a chromosome was set to 0.3**α*. Therefore, the number of each link in the initial population was 128*0.3**α*. For example, the expected number of links with a prior probability of 1 in the initial population was approximately 38. Unfortunately, our prior information does not include the directionality of gene regulation (*k*). To deal with this blindness, our GA initialization procedure assigned *k*=1 for half of the links and *k*=-1 for the other half. When α=1.0, 19 positive links and 19 negative links were created in the whole population. For links with a low α, it is quite possible that there is no occurrence in the population (e.g., < 2 links with α=0.1). Therefore, all the links in the prior table with a probability greater than zero were forced to have at least four expectations of occurrence in the initial population. In addition to the links from the prior table, a certain number of random links were added to the chromosomes. The random links were created based on the Poisson distribution with *λ*=1/10000. Since the number of all possible links in a chromosome is *N*(N-1)*, where number of genes *N*=13047, the expected number of randomly generated links, namely *λ*N*(N-1)*, equals to 17,021.

**GA network evaluation**

We used the gene expression profile consisting of 13,047 genes across 1,386 breast cancer samples. As described above, the PAM method was used to classify samples into three groups in which the gene of question is overexpressed, underexpressed or neutrally expressed. This dataset was used to generate a pre-calculated score table based on the conditional probability. This score table was used as the GA landscape for the evaluation process. For a pair of gene A and B, the expression data provide co-expression patterns across 1,386 instances. Some instances may indicate the overexpression of both A and B while others may support the underexpression of A and the overexpression of B. The conditional probabilities can be calculated by observing all instances as follows.

Positive regulation score (PRS) for A -> B

$$PRS[A][B] = P(B is overexpressed | A is overexpressed) - P(B is overexpressed)$$

$$+ P\left( B is underexpressed \right|A is underexpressed) - P(B is underexpressed)$$

Negative regulation score (NRS) for A -> B

$$NRS[A][B] = P(B is underexpressed | A is overexpressed) - P(B is underexpressed)$$

$$+ P(B is overexpressed | A is underexpressed) - P(B is overexpressed)$$

Overall regulation score (ORS) for A -> B

$$ORS[A][B] = P(B is over or underexpressed | A is over or underexpressed)$$

$$- P(B is over or underexpressed)$$

$$= PRS[A][B] + NRS[A][B]$$

From this landscape, we can calculate the score of each link in a chromosome as follows.

If a link E predicts that gene X regulates gene Y positively,

$$Score(E) = ORS[A][B] + PRS[A][B]$$

If a link E predicts that gene X regulates gene Y negatively,

$$Score(E) = ORS[A][B] + NRS[A][B]$$

The fitness score of a chromosome C is calculated as

$$\mathrm{Score}\left( C \right)= \left( \sum_{\forall link E\subseteq C} \mathrm{Score}\left( E \right)+\sum_{\forall link E2\subseteq C\times C} \mathrm{Score}\left( E2 \right) \right)\times Compensation$$

where the compensation function is defined as

$$Compensation = \frac{E(|C|)}{E(|C|)+|C|}$$

where $\left| C \right|= number of links in C and E(|C|) = expected number of links in C$.

The expected number of links equals to the sum of all the probabilities in the prior table. The compensation function is critical because the fitness score is highly dependent on the number of links in a chromosome. In general, a chromosome with more regulatory links tends to have a higher fitness score. When the GA is implemented without compensation, the number of links in a population increases according to the number of generations during the evolutionary process. An adequate compensation function is required to maintain a proper number of links in the selected optimal networks. Our scoring function takes into account two-hop regulation links. Two-hop links mean gene pairs reachable with two hops. For example, if the link A -> B and link B -> C are present in a chromosome, then the link A -> C is a two-hop link in this chromosome. The sign of the link (the directionality of gene regulation, namely positive or negative) for two-hop links was determined by the multiplication of the two connecting one-hop links. If A -> B is negative and B -> C is also negative, then A -> C becomes a positive two-hop link. It is expected that the possible number of two-hop links is proportional to the square number of links. Therefore, the asymptotic time complexity of GA evaluation is O(|C|^2^) where |C| is the number of links in the chromosome C.

**GA network selection**

The selection process of the GA makes parts of the chromosomes become either more or less frequent in the population. A chromosome with a higher fitness score is allowed to pass down part of its information at a higher rate. Selection is carried out based on the fitness score obtained at the evaluation step. Firs, the chromosomes in the population are sorted into four groups (elite, good, normal, and poor) according to their fitness score. Next, the first chromosome in the elite group is crossed with the first chromosome in the poor group according to the crossover operation as described below. One of the daughter chromosomes generated by the crossover is randomly chosen to replace the first chromosome of the poor group. The first chromosome of the elite group is not substituted and remains until the next generation. This is repeated for all the chromosomes in the elite and poor group. As a result, the elite chromosomes transfer on average 150% of their information to the next generation whereas the poor chromosomes pass on only 50% of their information. The crossover between the good and normal groups is carried out in a different fashion than that between the elite and poor groups. After the crossing of the first chromosome in the good group with the first chromosome in the normal group by means of the same crossover operation, the two daughter chromosomes replace the first chromosomes of the good and normal groups. This type of crossover is repeated for all the chromosomes in the good and normal group. As a result, the members of the good and normal groups pass down 100% of their information overall to the next generation. In this manner, the chromosomes with a high fitness score propagate their variants in the population while those with a poor fitness score gradually vanish. After the crossover is finished, some links in the individual chromosomes are changed by the mutation operation. We used three types of mutation operators in our GA process: 1) deleting a randomly selected link from the chromosome, 2) adding a randomly created link to the chromosome, and 3) changing the regulatory sign of randomly selected links.

**GA termination**

The whole process of evaluation, selection, crossover and mutation was repeated for 20,000 generations, after which the links that were retained in at least 40% of the populations were chosen. The termination parameters were determined empirically based on repeated observations. The rate of evolution (the overall increase of the fitness score) reduced considerably after 10,000 generations, at which only about 1% of the links were changing. In an additional test, we found that only 0.1% of the links changed on average between the 20,000th generation through the 100,000th generation. As network size grows, optimizing network structure becomes less feasible and only local optimal solutions can be obtained. We repeated our GA for 1,000 times to generate seed networks for further optimization by the MCMC-based algorithm (9–14). The final seed networks were allowed to contain loop structures except self-loops.

**Computational performance of GA**

Starting with 3,328,575 lines of prior information and 13,047 genes, our GA created about 210,000 links for each chromosome in the initial population. The number of links decreased gradually during the evolutionary operation of the GA leading to approximately 130,000 links in the optimized chromosomes on average. Across the 1,000 final networks, most links were rarely found while some were commonly present. At the threshold of 30% conservation, the number of the conserved links did not vary significantly when summing 100 networks, 300 networks or 1,000 networks. However, at a lower threshold (e.g. 20% conservation), the number of links in the combined network was dependent on the number of the combining seed networks. We observed that the running time was highly dependent on the amount of prior information and gene co-expression data. Most of the computation time was spent during the evaluation process. The total CPU time of our GA run for the 20,000-generation evolution of 128 chromosomes in one population was 292,300 seconds per i5-3570 CPU (3.40 GHz). Optimizing one seed network through the MCMC algorithm measured 61,350 seconds on the identical computer platform. We ran our GA for 1,000 populations consisting of 128 chromosomes, resulting in 1,000 suboptimal networks evolutionarily selected from each population. The 1,000 networks were fed into another round of learning procedure that utilized an MCMC-based algorithm. The total CPU time for this is, therefore, 292,300*1,000+61,350*1,000 seconds. This amounts to testing 128*1,000 seed networks purely through the MCMC-based learning procedure, which is estimated to cost 61,350*1,000*128 seconds. Therefore, our GA-MCMC approach is assessed to be 22.2 times faster than the pure MCMC approach for the given parameters. Using one hundred parallel processors, our GA-MCMC method would take 40 days while the pure MCMC would require 2.5 years.

**Processing of public mutation or SNP data**

We collected 112 breast cancer GWAS SNPs reported at the GWAS catalog (15) (http://www.genome.gov/gwastudies/). We then used the SNAP tool (16) to include polymorphic loci in linkage disequilibrium (LD) with the GWAS SNPs at *r*^2^=1.0 and the distance limit of 500 kb based on the 1000 Genome Pilot and HapMap 3 database, leading to a total of 519 breast cancer risk loci. We first searched our global network for their targeting genes along with associated regulators as evidenced by our TF prior information. We then searched for the genes that are genetically associated in cis or in trans with the risk loci by interrogating previous eQTL maps generated in the context of breast cancer (8), lymphoblastoid cells (17), brain (10, 18, 19), lung (20), and liver (21). We also included the genes reported to the GWAS catalogue based on their proximity to the associated risk SNP and their functional role in relation with breast cancer development. A total of 90 risk genes were identified. Non-coding mutations occurring in breast cancer were obtained from the whole-genome sequencing of 21 patients (22, 23). A total of 105 driver or significantly mutated genes were obtained from previous breast cancer exome sequencing studies in which driver coding mutations were predicted based on their recurrence patterns (24–26) . The driver genes, in particular, were defined as containing mutations that were discovered and confirmed in a large number of cases to be recorded in the Catalogue Of Somatic Mutations in Cancer (COSMIC) database. The significantly mutated genes were identified as having passed statistical tests based on the above three breast cancer sequencing results. Both categories were used to define the driver genes in our analysis. Twenty-nine of the 105 driver genes were expected to function as a TF according to a previous study (27) and we named them coding driver factors.

**References**

1. Matys,V., Fricke,E., Geffers,R., Gössling,E., Haubrock,M., Hehl,R., Hornischer,K., Karas,D., Kel,A.E., Kel-Margoulis,O. V, *et al.* (2003) TRANSFAC: transcriptional regulation, from patterns to profiles. *Nucleic Acids Res.*, **31**, 374–378.

2. Grant,C.E., Bailey,T.L. and Noble,W.S. (2011) FIMO: scanning for occurrences of a given motif. *Bioinformatics*, **27**, 1017–1018.

3. Bailey,T.L., Boden,M., Buske,F.A., Frith,M., Grant,C.E., Clementi,L., Ren,J., Li,W.W. and Noble,W.S. (2009) MEME SUITE: tools for motif discovery and searching. *Nucleic Acids Res.*, **37**, W202–W208.

4. Neph,S., Stergachis,A.B., Reynolds,A., Sandstrom,R., Borenstein,E. and Stamatoyannopoulos,J. a (2012) Circuitry and dynamics of human transcription factor regulatory networks. *Cell*, **150**, 1274–86.

5. Li,G., Ruan,X., Auerbach,R.K., Sandhu,K.S., Zheng,M., Wang,P., Poh,H.M., Goh,Y., Lim,J., Zhang,J., *et al.* (2012) Extensive promoter-centered chromatin interactions provide a topological basis for transcription regulation. *Cell*, **148**, 84–98.

6. Fullwood,M.J., Liu,M.H., Pan,Y.F., Liu,J., Xu,H., Mohamed,Y. Bin, Orlov,Y.L., Velkov,S., Ho,A., Mei,P.H., *et al.* (2009) An oestrogen-receptor-alpha-bound human chromatin interactome. *Nature*, **462**, 58–64.

7. Phillips,J.E. and Corces,V.G. (2009) CTCF: master weaver of the genome. *Cell*, **137**, 1194–1211.

8. Curtis,C., Shah,S.P., Chin,S.-F., Turashvili,G., Rueda,O.M., Dunning,M.J., Speed,D., Lynch,A.G., Samarajiwa,S., Yuan,Y., *et al.* (2012) The genomic and transcriptomic architecture of 2,000 breast tumours reveals novel subgroups. *Nature*, **486**, 346–352.

9. Zhu,J., Wiener,M.C., Zhang,C., Fridman,A., Minch,E., Lum,P.Y., Sachs,J.R. and Schadt,E.E. (2008) Increasing the power to detect causal associations by combining genotypic and expression data in segregating populations. *PLoS Comput. Biol.*, **3**, e69.

10. Zhang,B., Gaiteri,C., Bodea,L.-G., Wang,Z., McElwee,J., Podtelezhnikov,A.A., Zhang,C., Xie,T., Tran,L., Dobrin,R., *et al.* (2013) Integrated systems approach identifies genetic nodes and networks in late-onset Alzheimer’s disease. *Cell*, **153**, 707–720.

11. Chen,Y., Zhu,J., Lum,P.Y., Yang,X., Pinto,S., MacNeil,D.J., Zhang,C., Lamb,J., Edwards,S., Sieberts,S.K., *et al.* (2008) Variations in DNA elucidate molecular networks that cause disease. *Nature*, **452**, 429–35.

12. Jostins,L., Ripke,S., Weersma,R.K., Duerr,R.H., McGovern,D.P., Hui,K.Y., Lee,J.C., Schumm,L.P., Sharma,Y., Anderson,C. a, *et al.* (2012) Host-microbe interactions have shaped the genetic architecture of inflammatory bowel disease. *Nature*, **491**, 119–24.

13. Zhu,J., Sova,P., Xu,Q., Dombek,K.M., Xu,E.Y., Vu,H., Tu,Z., Brem,R.B., Bumgarner,R.E. and Schadt,E.E. (2012) Stitching together multiple data dimensions reveals interacting metabolomic and transcriptomic networks that modulate cell regulation. *PLoS Biol.*, **10**, e1001301.

14. Zhu,J., Zhang,B., Smith,E.N., Drees,B., Brem,R.B., Kruglyak,L., Bumgarner,R.E. and Schadt,E.E. (2008) Integrating large-scale functional genomic data to dissect the complexity of yeast regulatory networks. *Nat. Genet.*, **40**, 854–861.

15. Hindorffa,L.A., Sethupathyb,P., Junkinsa,H.A., Ramosa,E.M., Mehtac,J.P., Collinsb,F.S. and Manolioa,T.A. (2009) Potential etiologic and functional implications of genome-wide association loci for human diseases and traits. *Proc. Natl. Acad. Sci. USA*, **106**, 9362–9367.

16. Johnson,A.D., Handsaker,R.E., Pulit,S.L., Nizzari,M.M., O’Donnell,C.J. and de Bakker,P.I. (2008) SNAP: a web-based tool for identification and annotation of proxy SNPs using HapMap. *Bioinformatics*, **24**, 2938–2939.

17. Pickrell,J.K., Marioni,J.C., Pai,A.A., Degner,J.F., Engelhardt,B.E., Nkadori,E., Veyrieras,J.B., Stephens,M., Gilad,Y. and Pritchard,J.K. (2010) Understanding mechanisms underlying human gene expression variation with RNA sequencing. *Nature*, **464**, 768–772.

18. Gibbs,J.R., Brug,M.P. van der, Hernandez,D.G., Traynor,B.J., Nalls,M.A., Lai,S.-L., Arepalli,S., Dillman,A., Rafferty,I.P., Troncoso,J., *et al.* (2010) Abundant Quantitative Trait Loci Exist for DNA Methylation and Gene Expression in Human Brain. *PLoS Genet.*, **6**, e1000952.

19. Myers,A.J., Gibbs,J.R., Webster,J.A., Rohrer,K., Zhao,A., Marlowe,L., Kaleem,M., Leung,D., Bryden,L., Nath,P., *et al.* (2007) A survey of genetic human cortical gene expression. *Nat. Genet.*, **39**, 1494–1499.

20. Hao,K., Bossé,Y., Nickle,D.C., Paré,P.D., Postma,D.S., Laviolette,M., Sandford,A., Hackett,T.L., Daley,D., Hogg,J.C., *et al.* (2012) Lung eQTLs to help reveal the molecular underpinnings of asthma. *PLoS Genet.*, **8**, e1003029.

21. Schadt,E.E., Molony,C., Chudin,E., Hao,K., Yang,X., Lum,P.Y., Kasarskis,A., Zhang,B., Wang,S., Suver,C., *et al.* (2008) Mapping the genetic architecture of gene expression in human liver. *PLoS Biol.*, **6**, e107.

22. Nik-Zainal,S., Van Loo,P., Wedge,D.C., Alexandrov,L.B., Greenman,C.D., Lau,K.W., Raine,K., Jones,D., Marshall,J., Ramakrishna,M., *et al.* (2012) The life history of 21 breast cancers. *Cell*, **149**, 994–1007.

23. Nik-Zainal,S., Alexandrov,L.B., Wedge,D.C., Van Loo,P., Greenman,C.D., Raine,K., Jones,D., Hinton,J., Marshall,J., Stebbings,L.A., *et al.* (2012) Mutational processes molding the genomes of 21 breast cancers. *Cell*, **149**, 979–993.

24. Banerji,S., Lawrence,M.S., Peng,S., Cibulskis,K., Sivachenko,A.Y., Ardlie,K.G., Rangel-Escareno,C., Sougnez,C., Auclair,D., Bautista-Pin˜a,V., *et al.* (2013) Sequence analysis of mutations and translocations across breast cancer subtypes. *Nature*, **486**, 405–409.

25. Stephens,P.J., Nik-Zainal,S., Butler,A., Tarpey,P.S., Martin,S., Cheverton,A., Davies,H., Varela,I., Gamble,J., Loo,P. Van, *et al.* (2012) The landscape of cancer genes and mutational processes in breast cancer. *Nature*, **486**, 400–404.

26. “The Cancer Genome Atlas Network” (2012) Comprehensive molecular portraits of human breast tumours. *Nature*, **490**, 61–70.

27. Ravasi,T., Suzuki,H., Cannistraci,C.V., Katayama,S., Bajic,V.B., Tan,K., Akalin,A., Schmeier,S., Kanamori-Katayama,M., Bertin,N., *et al.* (2010) An atlas of combinatorial transcriptional regulation in mouse and man. *Cell*, **140**, 744–52.
